# Minocycline protects against microgliopathy in a *Csf1r* haplo-insufficient mouse model of adult-onset leukoencephalopathy with axonal spheroids and pigmented glia (ALSP)

**DOI:** 10.1186/s12974-023-02774-1

**Published:** 2023-05-31

**Authors:** Xin Li, Banglian Hu, Xiaoyan Guan, Ziwei Wang, Yuhang Zhou, Hao Sun, Xian Zhang, Yanfang Li, Xiaohua Huang, Yingjun Zhao, Xin Wang, Huaxi Xu, Yun-Wu Zhang, Zhanxiang Wang, Honghua Zheng

**Affiliations:** 1 Xiamen Key Laboratory of Brain Center, The First Affiliated Hospital of Xiamen University, Fujian Provincial Key Laboratory of Neurodegenerative Disease and Aging Research, Institute of Neuroscience, School of Medicine, Xiamen University, Xiamen, 361102 Fujian China; 2 Basic Medical Sciences, School of Medicine, Xiamen University, Xiamen, 361102 Fujian China; 3 State Key Laboratory of Cellular Stress Biology, Xiamen University, Xiamen, 361102 China; 4 Department of Neurosurgery, Xiamen Key Laboratory of Brain Center, The First Affiliated Hospital of Xiamen University, Xiamen, 361102 China

**Keywords:** CSF1R, Microglia, Microgliopathy, Minocycline, ALSP

## Abstract

**Background:**

Mutations in colony-stimulating factor 1 receptor (CSF1R) are known to cause adult-onset leukoencephalopathy with axonal spheroids and pigmented glia (ALSP), which has been recently demonstrated as a primary microgliopathy characterized by cognitive impairment. Although the molecular mechanism underlying CSF1R-mediated microgliopathy remains unclear, therapeutic strategies have generally targeted modulation of microglial function. In particular, the microglial inhibitor, minocycline, has been shown to attenuate learning and memory deficits in several neurodegenerative diseases. The objectives of this study were to investigate the pathogenic mechanisms underlying ALSP and to explore the therapeutic effects of minocycline in an in vivo model of ALSP. We hypothesized that inhibiting microglial activation via minocycline could reverse the behavior and pathological defects in ALSP model mice.

**Methods:**

We generated a *Csf1r* haploinsufficiency mouse model of ALSP using CRISPR/Cas9 genome editing and conducted electrophysiological recordings of long-term potentiation (LTP) and behavioral tests to validate the recapitulation of clinical ALSP characteristics in 8- to 11-month-old mice. RNA-sequencing was used to explore enriched gene expression in the molecular pathogenesis of ALSP. Microglial activation was assessed by immunofluorescent detection of Iba1 and CD68 in brain sections of male ALSP mice and pro-inflammatory activation and phagocytosis were assessed in *Csf1r*^+/−^ microglia. Therapeutic effects were assessed by behavioral tests, histological analysis, and morphological examination after four weeks of intraperitoneal injection with minocycline or vehicle control in *Csf1r*^+/−^ mice and wild-type control littermates.

**Results:**

We found that synaptic function was reduced in LTP recordings of neurons in the hippocampal CA1 region, while behavioral tests showed impaired spatial and cognitive memory specifically in male *Csf1r*^+/−^ mice. Increased activation, pro-inflammatory cytokine production, and enhanced phagocytic capacity were also observed in *Csf1r*^+/−^ microglia. Treatment with minocycline could suppress the activation of *Csf1r*^+/−^ microglia both in vitro and in vivo. Notably, the behavioral and pathological deficits in *Csf1r*^+/−^ mice were partially rescued by minocycline administration, potentially due to inhibition of microglial inflammation and phagocytosis in *Csf1r*^+/−^ mice.

**Conclusions:**

Our study shows that CSF1R deficiency results in aberrant microglial activation, characterized by a pro-inflammatory phenotype and enhanced phagocytosis of myelin. Our results also indicate that microglial inhibition by minocycline can ameliorate behavioral impairment and ALSP pathogenesis in CSF1R-deficient male mice, suggesting a potential therapeutic target for CSF1R-related leukoencephalopathy. Collectively, these data support that minocycline confers protective effects against CSF1R-related microgliopathy in male ALSP model mice.

**Supplementary Information:**

The online version contains supplementary material available at 10.1186/s12974-023-02774-1.

## Introduction

Adult-onset leukoencephalopathy with axonal spheroids and pigmented glia (ALSP), a leukodystrophy characterized by dementia with neuropsychiatric deficits, is considered a single clinicopathologic entity of familial pigmented orthochromatic leukodystrophy (POLD) and hereditary diffuse leukoencephalopathy with axonal spheroids (HDLS), both of which are caused by autosomal dominant mutations in the *colony-stimulating factor 1 receptor* (*CSF1R*) gene [1–4].

CSF1R is mainly expressed on microglia in the brain [5], and disruption of CSF1R function results in primary microgliopathy in ALSP [1]. By contrast, dysfunction of microglia-related proteins, such as CSF1R, DAP12 and TREM2, USP18, and IRF8, is associated with secondary microgliopathy in a diverse set of neurological diseases, including Alzheimer’s disease (AD), amyotrophic lateral sclerosis (ALS), Parkinson’s disease (PD) and frontal temporal dementia (FTD) diseases [6–9]. Chitu et al. demonstrated that *Csf1r* heterozygosity is necessary and sufficient for development of the behavioral and neurodegenerative clinical characteristics observed in ALSP patients [10], and subsequent studies showed that a reduction of CSF1R specifically in microglia is sufficient to confer ALSP [11]. Microgliosis has been documented in both *Csf1r*^+/−^ mice and *Cx3Cr1*^*Cre/*+^;*Csf1r*^*fl/*+^ mice [10, 11]. Another recent study showed that treatment with the CSF1R inhibitor, PLX5622, to eliminate microglia in adult *Csf1r*^+/−^ mice could prevent microglial dyshomeostasis and attenuate the pathological phenotypes of *Csf1r*^+/−^ mice [12]. These studies indicate that microglial dyshomeostasis is a driver of ALSP pathogenesis, collectively supporting the possibility that modulating microglial homeostasis could reverse the pathogenesis of ALSP.

Here, in this study, we conducted long-term potentiation (LTP) assays that showed deficiency for one *Csf1r* allele results in early impairment of synaptic plasticity in the mouse brain, as well as defects in spatial and cognitive memory in *Csf1r*^+/−^ ALSP model mice, and furthermore, these deficits were specific to male mice. Subsequent investigation confirmed the aberrant activation of microglia in the *Csf1r*^+/−^ male mice. We then examined whether the purported "microglia inhibitor", minocycline, a semi-synthetic tetracycline derivative approved by the FDA more than 30 years ago to treat chronic inflammation and neurotoxicity [13–16], showed any therapeutic effects in alleviating microglial dysfunction in ALSP model mice. We found that treatment with minocycline inhibits the activation and inflammatory factor production by *Csf1r*^+/−^ microglia in vitro and in vivo. Notably, chronic administration of minocycline partially alleviated behavioral deficits and pathologies in 8-month-old *Csf1r*^+/−^ male mice. Our study provides a potential therapeutic strategy for ALSP by modulating microglial homeostasis in patients with CSF1R variants at high risk of ALSP pathogenesis.

## Materials and methods

### Reagents

Minocycline (Hydrochloride-CAS 13614-98-7-Calbiochem) was purchased from Sigma Aldrich (M9511; Oakville, ON, Canada). Anti-VGLUT1(RRID: AB_2797887), and anti-PSD95 (RRID: AB_561221) were all bought from Cell Signaling Technology. Anti-CSF1R (RRID: AB_776253), anti-Myelin Basic Protein (RRID: AB_297797), anti-α-Tubulin (RRID: AB_869989), anti GAPDH (RRID: AB_2630358), goat anti-rabbit IgG (H + L) (RRID: AB_10371940) and Alexa Fluor 647 donkey anti-rat IgG H&L (RRID: AB_150155) were all from Abcam. Anti-CSF1R (RRID: AB_884158) and granulocyte–macrophage colony-stimulating factor (GM-CSF) were from R&D Systems. Rabbit anti-Iba1 (RRID: AB_2687911) was purchased from Wako and rat anti-mouse CD68 (RRID: AB_322219) was from Bio-Rad. Alexa Fluor 488-conjugated goat anti-rabbit secondary antibody (RRID: AB_10374301) was bought from Thermo Fisher Scientific. DAPI (D9542) and poly-L-lysine (PLL, P6282) were purchased from Sigma Aldrich. TRIzol (CW05800s) and 100 × phosphatase inhibitor (CW2383S) were from CWBIO. ReverTra Ace qPCR RT Master Mix (R323-01) and HamQ Universal SYBR qPCR Master Mix (q711-03) were all from Vazyme. DMEM (11965126) and 1% penicillin/streptomycin (15140122) were all bought from Gibco. Fetal bovine serum (FBS, HK026) was from NTC. RIPA buffer (AR009550) and BCA Protein Assay Kit (AR0197) were from BOSTER. 50 × protease inhibitor (00329505) was purchased from Roche. Super ECL Detection Reagents (36208ES76) were from Yeasen. Fluoresbrite® YG Carboxylate Microspheres (1.00 µm, 15,702) was bought from Polyscience. Biotin-labeled antibodies and anti-streptomycin peroxidase (Kit-9270) and DAB Kit (DAB0031, 20 ×) were all from MXB. The hematoxylin solution (G1120) was from Solarbio.

### Animals and treatments

CSF1R heterozygous (*Csf1r*^+/−^; C57BL/6) mouse was generated using the CRISPR/Cas9 gene-editing system. CSF1R homozygous (HO, *Csf1r*^−/−^) mouse, CSF1R heterozygous (HE, *Csf1r*^+/−^) mouse and CSF1R wild-type (WT, *Csf1r*^+/+^) mouse were acquired by self-matching of *Csf1r*^+/−^ mouse. All mice were bred in the Xiamen University Laboratory Animal Center. Eight-month-old *Csf1r*^+*/*+^ or *Csf1r*^+/−^ mice were intraperitoneally (i.p.) injected with minocycline (Sigma Aldrich) for one month at a dose of 50 mg/kg/day [17, 18]. The detailed information (number, sex, and age) of mice used in each behavioral experiment is shown in Additional file 1: Table S1.

### Primary microglia culture and treatment

Primary microglia were isolated and cultured as described previously [19]. Briefly, brains from newborn pups were obtained and dissected. Mixed cells were suspended in DMEM supplemented with 10% FBS and cultured in 175 cm^2^ flasks (Nunc, 159910-ROS) coated with poly-L-lysine (PLL, P6282, Sigma Aldrich). The next day, media were replaced with fresh DMEM supplemented with 10% FBS and 25 ng/mL granulocyte–macrophage colony-stimulating factor (GM-CSF, R&D Systems). Microglia were harvested one week later by shaking the flasks at the speed of 220 rpm for 20 min. Cells were then cultured at a density of 1.5 × 10^6^ cells/well or 7 × 10^5^ cells/well in 6-well or 12-well plates and treated with or without 0.02 μM minocycline for 12 h for subsequent experiments according to the previous study [20].

### RNA sequencing and analysis

RNA-sequence was performed by Illumina HiSeq™ 2500/4000 and 5 G reads per sample were obtained on average (Gene Denovo Biotechnology Co., Ltd., Guangzhou, China). Detailed gene set enrichment analysis and weighted gene co-expression network analysis (WGCNA) were conducted according to previous literature [21, 22]. Data of all genes from all samples were calculated according to log2 (FPKM + 1). DESeq2 and edgeR software was used to analyze the differentially expressed genes (DEGs). The DEGs were selected as | log2(fold change) |> 1 and *P* value < 0.05 and were colored red for upregulated genes and blue for downregulated genes. These DEGs were mapped to Gene Ontology (GO) terms, molecular function (MF), cellular component (CC), and biological process (BP), in GO database. Significantly enriched GO terms of DEGs were examined and compared to the genome background through a hypergeometric test of DEGs. Similarly, significantly enriched pathways of DEGs were analyzed with Kyoto Encyclopedia of Genes and Genomes (KEGG) pathway database. Data of samples from 9-month-old *Csf1r*^+/+^ or *Csf1r*^+/−^ mice with or without minocycline treatment (*n* = 4 per group) were used for WGCNA. Gene co-expression networks were constructed with weighted expression correlations (soft power = 8). Hierarchical cluster analysis was carried out and the clustering results were segmented according to the standards (minModuleSize = 100, detectCutHeight = 0.95) to obtain different gene modules. The bioinformatic analyses were all performed using Omicsmart, an online platform for transcriptome data analysis (https://www.omicsmart.com).

### Western blot

Primary microglia or brain tissues were harvested and total proteins were extracted using RIPA buffer containing protease and phosphatase inhibitors. Equal amounts of proteins (20 μg for cell lysates, 50 μg for tissue lysates) were loaded into an 8% SDS-PAGE and transferred onto the PVDF membranes (Millipore, IPVH00010). The membranes were sequentially incubated with primary or secondary antibodies. Proteins were then visualized using Super ECL Detection Reagents and the bands were quantified using ImageJ Launcher (Broken Symmetry Software, 1.4.3.67).

### RNA extraction and quantitative real-time PCR (qRT-PCR)

Total RNA was extracted using TRIzol reagent and 1 μg RNA was reverse-transcribed into complementary DNA (cDNA) using ReverTra Ace qPCR RT Master Mix. Target genes were amplified using HamQ Universal SYBR qPCR Master Mix on the LightCycler 480 (Roche, Mannheim, Germany). The mRNA level of the target gene was calculated by the relative amount of the target gene ratio to those of the internal control *Actb* (2^−ΔΔCT^). The primer sequences are shown in Additional file 2: Table S2.

### IL-1β and TNF-α protein assay

Protein levels of IL-1β and TNF-α in the supernatant of mouse brain lysate or conditioned medium from primary microglia were measured according to the previous methods [23]. In brief, diluted samples were analyzed on the AST-Sc-Lite (a fully auto single-molecule detection machine supplied by Suzhou AstraBio Technology Co., Ltd.). The sample (25 μL) was incubated with Reagent 1 (mainly comprised 0.1 mg/mL magnetic beads coated with capture antibodies and protecting reagents) for 6 min. The samples were then incubated for 4 min under 40°C with Reagent 2 (mainly comprised mouse IL-1β or TNF-α detection antibodies labeled with single-molecule imagine fluorophores supplied by AstraBio). Magnetic beads labeled with fluorophores in the mixtures were absorbed by a magnet. Finally, the single-molecule fluorescent signals were taken and analyzed by the machine. IL-1β and TNF-α protein concentrations were calculated according to the standard curve.

### Transmission electron microscopy (TEM)

Mice were anesthetized with 5% chloral hydrate and brain tissues were acquired and fixed with the electron microscope fixative at 4℃ overnight. The corpus callosum was dissected and shaped into a trapezoidal section, fixed in osmium acid, followed by ethanol dehydration and uranium-saturated solution with Leica EM TP (Leica, Germany), and embedded with Spurr’s resin. Images were captured by transmission electron microscope Hitachi HT-7800 (Japan). Demyelination was analyzed by calculating the G-ratio of myelin within at least 8 to 10 fields per sample. The G-ratio is quantified by calculating the ratio of the axonal diameter/the myelinated fiber diameter. At least 100 myelinated axons per high-power field (HPF) in each mouse were randomly analyzed and calculated. The number of synapses per HPF of at least 10 visual fields from 5 mice/genotype was counted.

### Immunofluorescence staining

Brain slices or primary microglia were prepared and fixed with 4% paraformaldehyde (PFA). After incubated with the mouse anti-Iba1 (1:200) and anti-CD68 (1:200) overnight at 4°C, slices or cells were then incubated with the Alexa Fluor 488-conjugated goat anti-rabbit secondary antibody (1:500) and Alexa Fluor 647 Donkey anti-Rat IgG H&L (1:500) for 1 h. *Z*-stack confocal images were captured with an Olympus FV1000MPE-B confocal microscope (Japan). The number of Iba1^+^ microglia was double-blindly counted by ImageJ software (Version 2006.02.01). The number of branches and Iba1^+^ CD68^+^ double-positive cells per HPF in the hippocampus from three to five mice were analyzed by Olympus FV10-ASW 4.0 Viewer software and the 3D microglial morphology was reconstructed by the Imaris software (Bitplane, Belfast, UK, version 9.2.0).

### Electrophysiological recording

The electrophysiological recording was performed as described previously [24]. Mice were anesthetized with isoflurane and the brains were then rapidly acquired and placed in ice-cold high-sucrose artificial cerebrospinal fluid (ACSF; 120 mM sucrose, 2.5 mM KCl, 10 mM MgSO_4_, 1.25 mM NaH_2_PO_4_, 26 mM NaHCO_3_, 10 mM d-glucose, 64 mM NaCl, and 0.5 mM CaCl_2_, pH 7.4, ~ 310 mOsm) bubbled with carbogen (95% O_2_ + 5% CO_2_). Brain slices (400 μm thick) were sectioned using a Leica VT1200S (Germany) vibratome, incubated in ACSF with low magnesium (3.5 mM KCl, 120 mM NaCl, 1.3 mM MgSO_4_, 10 mM d-glucose, 1.25 mM NaH_2_PO_4_, 26 mM NaHCO_3_, and 2.5 mM CaCl_2_ (pH 7.4), ~ 300 mOsm) bubbled with carbogen (95% O_2_ + 5% CO_2_) at 32 °C for 1 h. Mouse LTP was recorded by Axon™ Digidata 1550 (USA) and Diaphragm clamp Amplifier (Axon CNS, Multiclamp 700B, USA). Schaffer peripheral inputs in CA3 were stimulated by Concentric Bipolar Electrode (FHC, CBARC75, Inc. Bowdoin, ME, USA). Field excitatory postsynaptic potential (fEPSP) of Schaffer lateral pathways in the CA1 was recorded with 800 kΩ-2 MΩ epoxy glass (Sutter Instrument, BF150-86–10, USA). After a 20-min stable baseline recording, LTP was induced by high-frequency stimulation (HFS, two trains of 100-Hz stimuli with an interval of 30 s), followed by continued recording for 60 min.

### Myelin sheath purification and phagocytosis assay

Myelin sheath was purified as previously described [25] and stored at − 80°C for further use. The pHrodo-conjugated myelin sheath was acquired by incubating the myelin sheath with Amine-Reactive pHrodo™ Dyes for 1 h at RT. Before use, the pHrodo-conjugated myelin sheath was suspended in DMEM to the final concentration of 1.2 mg/mL. Primary microglia were seeded in chamber slides (1 × 10^5^ cells per well) and treated with phagocytic beads (Alexa Fluor 488, 1:15,000), or pHrodo myelin sheath (Alexa Fluor 568, 1:1000) for 3 h. Cells were then fixed with 4% PFA and subjected to immunostaining with the rabbit anti-Iba1 (1:200) at 4°C overnight, following stained with donkey anti-rabbit IgG H&L (1:500, Alexa Fluor 568) or Goat anti-rabbit IgG H&L (1:500, Alexa Fluor 488). Images were captured with an Olympus FV1000MPE-B confocal microscope (Japan). The number of Iba1^+^ cells co-localized with phagocytic beads or pHrodo myelin sheath was manually counted. Meanwhile, the fluorescence area of pHrodo myelin sheath within each Iba1^+^ cell was calculated and analyzed using the "ROI (region of interest) " by setting analysis parameters as “area”, “mean gray value”, “limit to threshold” in the "measure" of ImageJ software (Version 2006.02.01). Four or five fields in each group from three independent experiments were analyzed.

### Behavior tests

Before behavioral experiments, all the mice were gently touched for 3 days to avoid stress responses and placed in the test chamber for more than 30 min to adapt to the environment. All the behavior tests were performed and analyzed using CleverSys TopScanLite animal behavior analysis system (USA).

### T-maze test

T-maze was used to evaluate the working memory of mice. The length of T-maze arms is 30 × 6 × 10 cm. At the beginning of the test, mice were placed in the initial arm, and the trajectory of mice was collected by Clever Sys system for 5 min [24]. The alternation triplet (%) within three arms of each mouse was analyzed.

### Novel object recognition test (NOR)

NOR was mainly used to evaluate the spatial alternation and working memory of mice [26]. The training was divided into three stages. In the training stage, each mouse was allowed to move freely in a box of 40× 40× 40 cm for 10 min to adapt to the environment. In the test stage (old object recognition training), two identical objects wrapped in the red paper were prepared and labeled as A or B, which were placed on two opposite angles, allowing the mice to explore freely. The test time was 10 min. In the novel object test stage, object B was replaced with C (new object) wrapped in yellow paper and marked as C. Each mouse was allowed to explore object A or C freely for 10 min. The time of exploring object A or C was recorded. Finally, the time ratio of the exploring object was analyzed and indicated as the new object recognition index (RI) = C/(A + C) × 100%.

### Open field test

The open-field test was performed according to previous reports [27]. Mice were allowed to explore freely for 10 min. The time and distance of spontaneous activity of mice in the center of the open field box were analyzed.

### Light–dark transition test

Light–dark transition test was used to test the anxiety-like behavior of mice, which was first described by Crawley and Goodwin (1980) [28]. The light–dark box was mainly composed of two different boxes (40 × 15× 10 cm), namely a black box and a white box. At the beginning of the test, mice were placed at the door of the black box with their head facing the white box and were free to explore for 10 min. The time staying in the white box was observed and analyzed.

### Sucrose preference test (SPT)

SPT was used to assess depression-like behavior in mice. The experiment is performed as previously described [27]. The sucrose preference test lasted for 2 days and consisted of two processes: the training process and the testing process. During the training process, all mice were given a bottle of 1% (W/V) sucrose in each cage during the first 24 h. During the test process, every mouse was placed in a separate cage containing one bottle of 1% (W/V) sucrose and one bottle of pure water. The position of the two bottles was changed after 6 h. Sucrose preference (%) is calculated and analyzed according to the following formula: 1% sucrose consumption/(1% sucrose consumption + pure water consumption) × 100%.

### Statistical analysis

Statistical analysis was performed in a double-blinded manner using GraphPad Prism Software (GraphPad Software, CA, USA; version 8.0.2). The unpaired two-tailed Student's* t*-test was used to compare two groups. One-way or two-way analysis of variance (ANOVA) post-Sidak’s multiple comparisons test was used for the comparison of more than two groups. Data were expressed as mean ± SEM. A *p*-value less than 0.05 was considered statistically significant.

## Results

### Impaired synaptic function in CRISPR/Cas9-based *Csf1r*^+/−^ mouse model

**Fig. 1:**
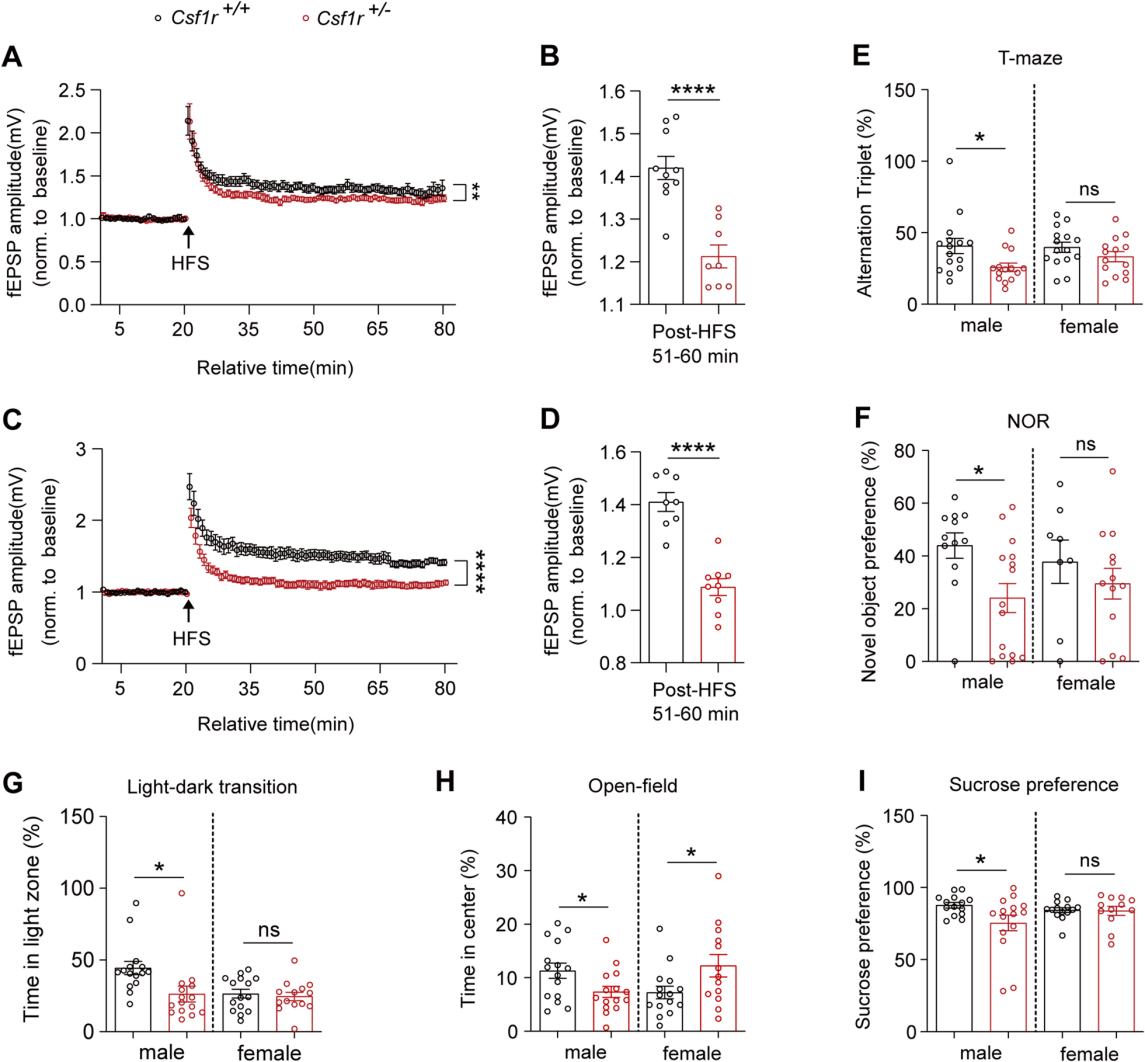
Impaired synaptic and cognitive function in *Csf1r*^+/−^ male mice. **A** Time series plots of evoked field potentials recorded in the CA1 area of hippocampal slices from 3- to 5-month-old *Csf1r*^+*/*+^ or *Csf1r*^+/−^ mice. HFS, high-frequency stimulation. **B** The average fEPSP amplitude over the last 10 min of LTP recording was quantified. *Csf1r*^+*/*+^ (*n* = 5 mice, 10 slices), *Csf1r*^+/−^ (*n* = 5 mice, 8 slices). **C** Time series plots of evoked field potentials recorded in the CA1 area of hippocampal slices from 10- to 11-month-old *Csf1r*^+*/*+^ or *Csf1r*^+/−^ mice. **D** The average fEPSP amplitude over the last 10 min of LTP recording was quantified. *Csf1r*^+*/*+^ (*n* = 5 mice, 8 slices), *Csf1r*^+/−^ (*n* = 5 mice, 9 slices). **E** Behavioral performance in T-maze test. The percentage of alternation triplets within 5 min was recorded and analyzed (*n* = 14–15 mice). **F** Novel object recognition (NOR). The preference ratio (time spent on the novel object / time spent on the old and novel object) was calculated: C/(A + C) × 100% (*n* = 8–15 mice). **G** Light–dark transition test. The ratio of time spent in the white box to the total 10 min experiment duration (*n* = 14–15 mice). **H** Behavioral performance in open-field test. The time spent in the center of the open field within 10 min was recorded and analyzed (*n* = 14–15 mice). **I** Sucrose preference test. Sucrose preference was analyzed by calculating the percentage of the final remaining amount of sucrose (*n* = 12–15 mice). Data are presented as means ± SEM. *P*-values were calculated using unpaired two-tailed Student’s *t*-test. *p < 0.05; **p < 0.01; ****p < 0.0001; ns, no significance

Previous studies have shown that memory loss is one of the initial symptoms presenting in ALSP patients [1, 29, 30]. To investigate whether deficiency for one *Csf1r* allele results in early impairment of synaptic plasticity, we generated *Csf1r* haploinsufficient mice using CRISPR/Cas9 genome editing (Additional file 7: Fig. S1A–H). We then examined the effects of *Csf1r* haploinsufficiency on the functional electrophysiological characteristics of synapses in the hippocampal CA1 brain region of *Csf1r*^+/−^ mice by evaluating LTP, a form of synaptic plasticity that contributes to learning and memory formation. LTP elicited by high-frequency stimulation (HFS) was significantly decreased in 3- to 5-month-old *Csf1r*^+/−^ male mice compared to that in wild-type (WT) littermates (Fig. 1A, B) and further substantially reduced in 10- or 11-month-old *Csf1r*^+/−^ male mice (Fig. 1C, D). However, the LTP elicited by HFS was not significantly affected in 8- or 9-month-old female mice (Additional file 7: Fig. S2A, B). Spontaneous alternation in T-maze tests showed that spatial working memory was also impaired in *Csf1r*^+/−^ male mice compared with their *Csf1r*^+/+^ male littermates (Fig. 1E). Evaluation of hippocampal recognition memory using NOR tests in these mice showed that *Csf1r*^+/−^ male mice spent less time exploring the novel object than that of their male *Csf1r*^+/+^ littermates (Fig. 1F), although no significant differences were observed in T-maze or NOR tests between *Csf1r*^+/−^ and *Csf1r*^+/+^ female mice. Light–dark transition tests showed clear anxiety-like behaviors (Fig. 1G) in these ALSP male mice, but not in control males or female mice. Anxiety or depression-like behaviors, validated by open-field tests (Fig. 1H) or sucrose preference tests (Fig. 1G–I), were also observed in those *Csf1r*^+/−^ male mice, but not in control males or female mice, which was consistent with those previous studies [10]. Taken together, these results demonstrated that *Csf1r* haploinsufficiency impairs synaptic function in addition to associated impairment of spatial and cognitive memory impairments in the *Csf1r*^+/−^ ALSP model mice.

### *Csf1r* haploinsufficiency results in an inflammatory and phagocytic microglial phenotype in vivo and in vitro

**Fig. 2:**
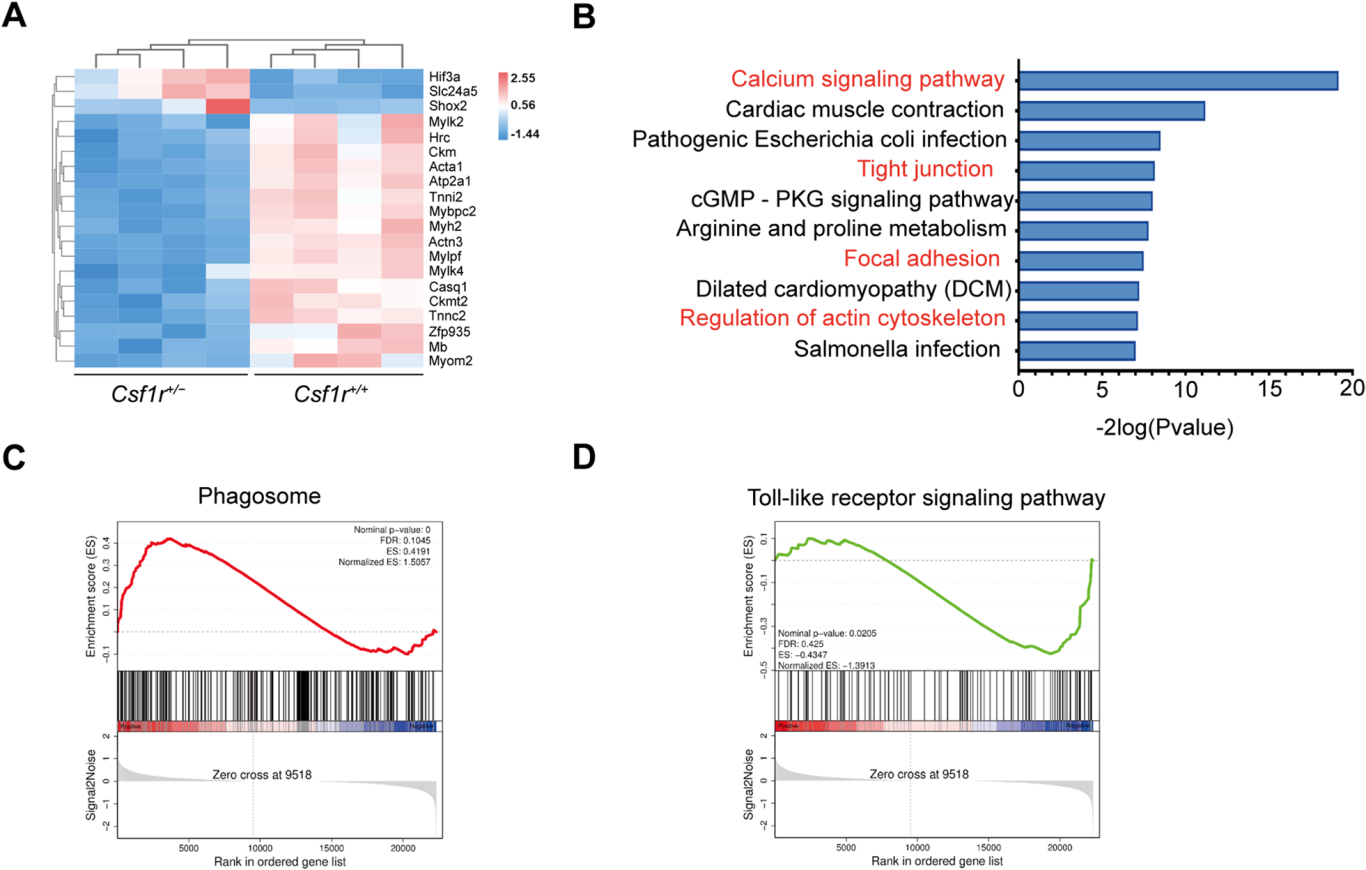
Transcriptomic differences in forebrain samples of *Csf1r*^+/+^ or *Csf1r*^+/−^ male mice. **A** Heatmap showing differentially expressed genes (DEGs) in 9-month-old cerebral brains from four *Csf1r*^+*/*+^ or *Csf1r*^+/−^ mice. The color key (from blue to red) of the *Z*-score value (− 1.44–2.25) indicates low to high expression levels. **B.** KEGG pathway analysis of DEGs showing enrichment for the phagosome-related pathways in the cortices of *Csf1r*^+/−^ mice. The top ten high confidence pathways and phagocytosis-associated pathways (red). **C**, **D** GSEA of DEGs in forebrain samples of *Csf1r*^+*/*+^ or *Csf1r*^+/−^ mice showing enrichment with upregulated gene sets in the phagosome pathway in CSF1R haploinsufficient mice (**C**) and enrichment with downregulated gene sets in the Toll-like receptor signal pathway (**D**)

Whole transcriptome RNA sequencing in forebrain tissue from *Csf1r*^+/+^ or *Csf1r*^+/−^ mice was performed to further explore the pathogenic mechanisms related to *Csf1r* haploinsufficiency. High-confidence differentially expressed genes (DEGs) from the two groups were examined by heatmap visualization (Fig. 2A, Additional file 3: Table S3) followed by Gene Ontology (GO) and KEGG analyses (Fig. 2B). The top ten KEGG pathways, classified by log2 (*P* value), were significantly enriched in genes related to calcium signaling pathway, cell junction-like tight junctions, and focal adhesion pathways. Additionally, gene set enrichment analysis (GSEA) showed noteworthy enrichment in phagosome pathway (Fig. 2C, genes listed in Additional file 4: Table S4) and toll-like receptor pathway (Fig. 2D, genes listed in Additional file 5: Table S5). These GSEA results showed that *CSF1R* haploinsufficiency leads to significant upregulation of phagosome-related genes (see heatmap with *Z*-score in Additional file 7: Fig. S3A), but down-regulation of the genes associated with toll-like receptor pathway (see heatmap with *Z*-score in Additional file 7: Fig. S3B), which indicates that *CSF1R* haploinsufficiency may affect microglial dyshomeostasis in the brain.

**Fig. 3:**
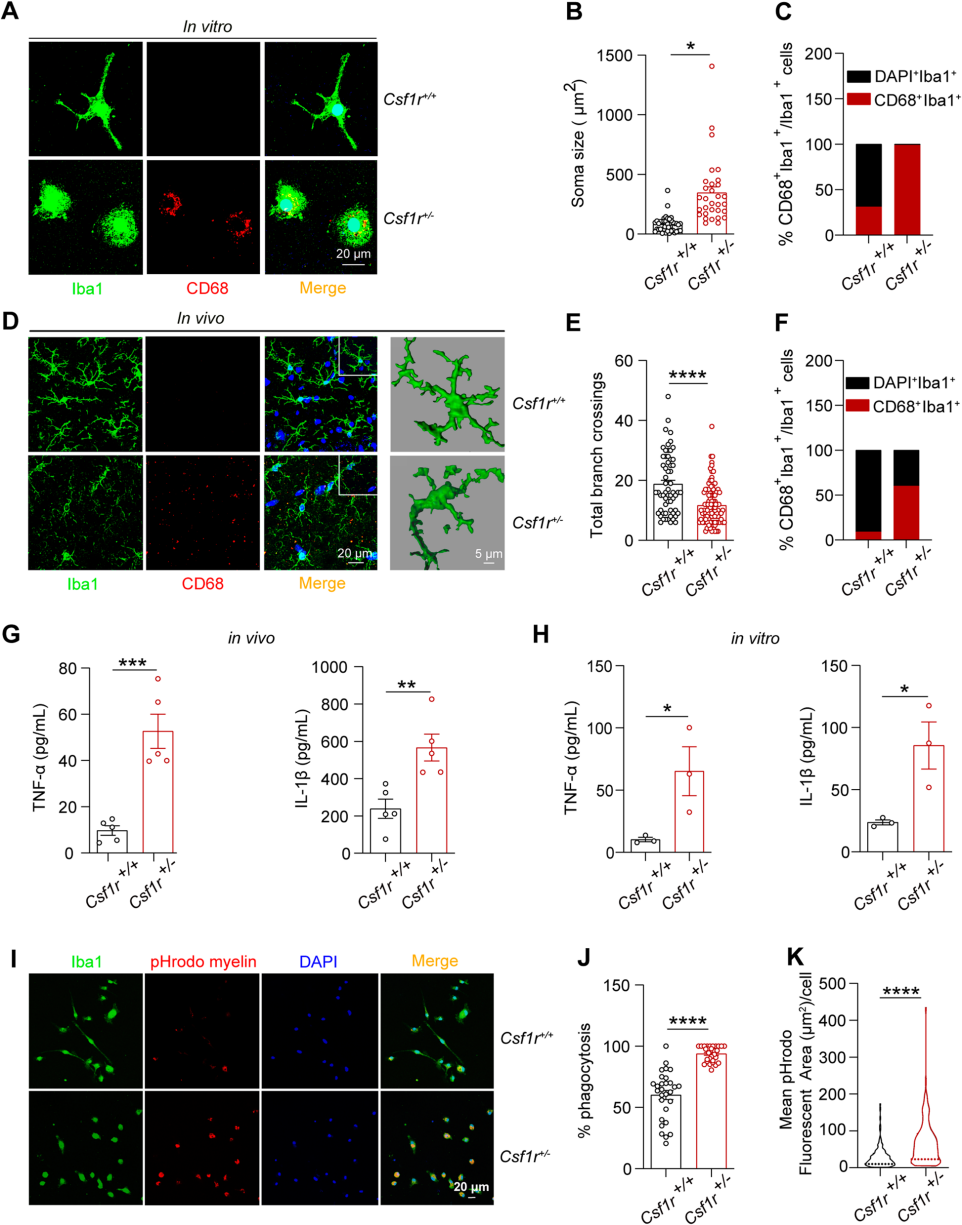
*Csf1r*^+/−^ microglia show an inflammatory and phagocytic phenotype in vivo and in vitro*.* **A** Representative Iba1 (green) and CD68 (red) immunofluorescent images of *Csf1r*^+*/*+^ and *Csf1r*^+/−^ primary microglia. Note the amoeboid-like morphology of *Csf1r*^+/−^ microglia. **B** Statistical analysis of soma size in *Csf1r*^+*/*+^ and *Csf1r*^+/−^ microglia (*n* = 3 independent experiments). **C** Statistical analysis of the percentage of Iba1 (green) and CD68 (red) double-positive (Iba1^+^CD68^+^) microglia relative to Iba1^+^ microglia per field (*n* = 3 independent experiments). **D** Representative Iba1 (green) and CD68 (red) immunofluorescent images of brain slices from *Csf1r*^+*/*+^ or *Csf1r*^+/−^ mice at the age of 10 to 14 months. Three-dimensional (3D) reconstructions of representative Iba1^+^CD68^+^ microglial cells in the white box. **E** Statistical analysis of process number in Iba1^+^ microglia in the hippocampus of 10- to 14-month-old *Csf1r*^+*/*+^ (*n* = 5 mice, 61 cells) and *Csf1r*^+/−^ (*n* = 6 mice, 107 cells) mice. **F** Statistical analysis of the percentage of Iba1^+^CD68^+^ microglial cells relative to the number of Iba1^+^ microglia in hippocampus samples of 10- to 14-month-old *Csf1r*^+*/*+^ (*n* = 5 mice) and *Csf1r*^+/−^ (*n* = 6 mice) mice. **G** TNF-α and IL-1β protein levels in forebrain samples of *Csf1r*^+*/*+^ or *Csf1r*^+/−^ mice (*n* = 5 mice). **H** TNF-α and IL-1β protein levels in *Csf1r*^+*/*+^ or *Csf1r*^+/−^ microglia (*n* = 3 independent experiments). **I** Representative images of Iba1^+^ microglia after phagocytosis of red fluorescent dye (pHrodo)-labeled myelin sheaths. **J** Quantification of the ratio of Iba1^+^ pHrodo myelin-engulfing (pHrodo^+^) microglia to total Iba1^+^ microglia (*n* = 3 independent experiments). **K** The phagocytic ability of microglia on myelin sheaths quantified by mean pHrodo fluorescent intensity in Iba1^+^ cells (*n* = 3 independent experiments). Data are presented as means ± SEM. *P*-values were calculated using unpaired two-tailed Student’s *t*-test. *p < 0.05; **p < 0.01; ***p < 0.001; ****p < 0.0001

To further accurately describe the dyshomeostatic state of microglia in ALSP, the morphology and activation of *Csf1r*^+/+^ or *Csf1r*^+/−^ microglia were then observed in vitro and in vivo. Microglial activation is accompanied by switching from a ramified to an amoeboid-like morphology with an enlarged cell body and retracted processes [31]. We first observed the morphology changes by assessing the soma size of primary cultured *Csf1r*^+/−^ microglia shown by Iba1 staining (Fig. 3A). We found that *Csf1r*^+/−^ microglia exhibited an amoeboid-like morphology with increased soma size (Fig. 3A, B). We also examined the expression of CD68, a lysosomal protein that indicates the phagocytic state of microglia. Notably, the number of Iba1 and CD68 double-positive cells (Iba1^+^CD68^+^) in *Csf1r*^+/−^ microglia was increased compared to that population in *Csf1r*^+/+^ microglia (Fig. 3A, C). Furthermore, to confirm these results, the morphology and activation of microglia in *Csf1r*^+/+^ or *Csf1r*^+/−^ mouse brains were also investigated. A significant reduction in branch crossings was observed in male *Csf1r*^+/−^ mouse brains compared to age-matched *Csf1r*^+/+^ controls (Fig. 3D, E), which indicated that *Csf1r* deficiency resulted in retracted microglial processes. Simultaneously, the Iba1^+^CD68^+^ cell number was substantially increased in brain sections from *Csf1r*^+/−^ male mice when compared to those in control littermates (Fig. 3D–F). Thus, *Csf1r*^+/−^ microglia exhibited an amoeboid morphology with a phagocytic state both in vitro and in vivo (Fig. 3A–F).

The inflammatory phenotype was also evaluated in *Csf1r*^+/−^ microglia by measuring the production of inflammatory factors. We first evaluated the mRNA levels of IL-1β and TNF-α in *Csf1r*^+/+^ or *Csf1r*^+/−^ microglia. In the *Csf1r*^+/−^ mouse brain samples, *Il-1β* mRNA levels were significantly higher, and *Tnf-α* trended non-significantly higher, than that in WT littermates (Additional file 7: Fig. S4A), while both were significantly elevated in cultured primary microglia from *Csf1r*^+/−^ brains compared to *Csf1r*^+/+^ primary microglia (Additional file 7: Fig. S4B). To further validate the inflammation production, single-molecule assays to detect IL-1β and TNF-α levels confirmed that the production of both proteins was increased in primary *Csf1r*^+/−^ microglia and forebrain samples of *Csf1r*^+/−^ mice compared to that in their *Csf1r*^+/+^ littermates (Fig. 3G–H).

We next examined the phagocytic capacity of *Csf1r*^+/−^ microglia compared to *Csf1r*^+/+^ controls. Although *Csf1r* haploinsufficiency did not affect the phagocytosis of microsphere beads (Additional file 7: Fig. S5A-B), we observed significantly increased phagocytic uptake of labeled myelin sheaths in *Csf1r*^+/−^ microglia (Fig. 3I, J) and an increased mean pHrodo fluorescent area per cell (Fig. 3K) in *Csf1r*^+/−^ microglia compared to those in *Csf1r*^+/+^ microglia, indicating that this enhancement in microglial phagocytic function appears to be myelin-specific.

These results collectively indicated that *Csf1r* haploinsufficiency leads to a pro-inflammatory and phagocytic activation state of microglia.

### Minocycline inhibits partially the activation of *Csf1r* haploinsufficient microglia in vivo and in vitro

**Fig. 4:**
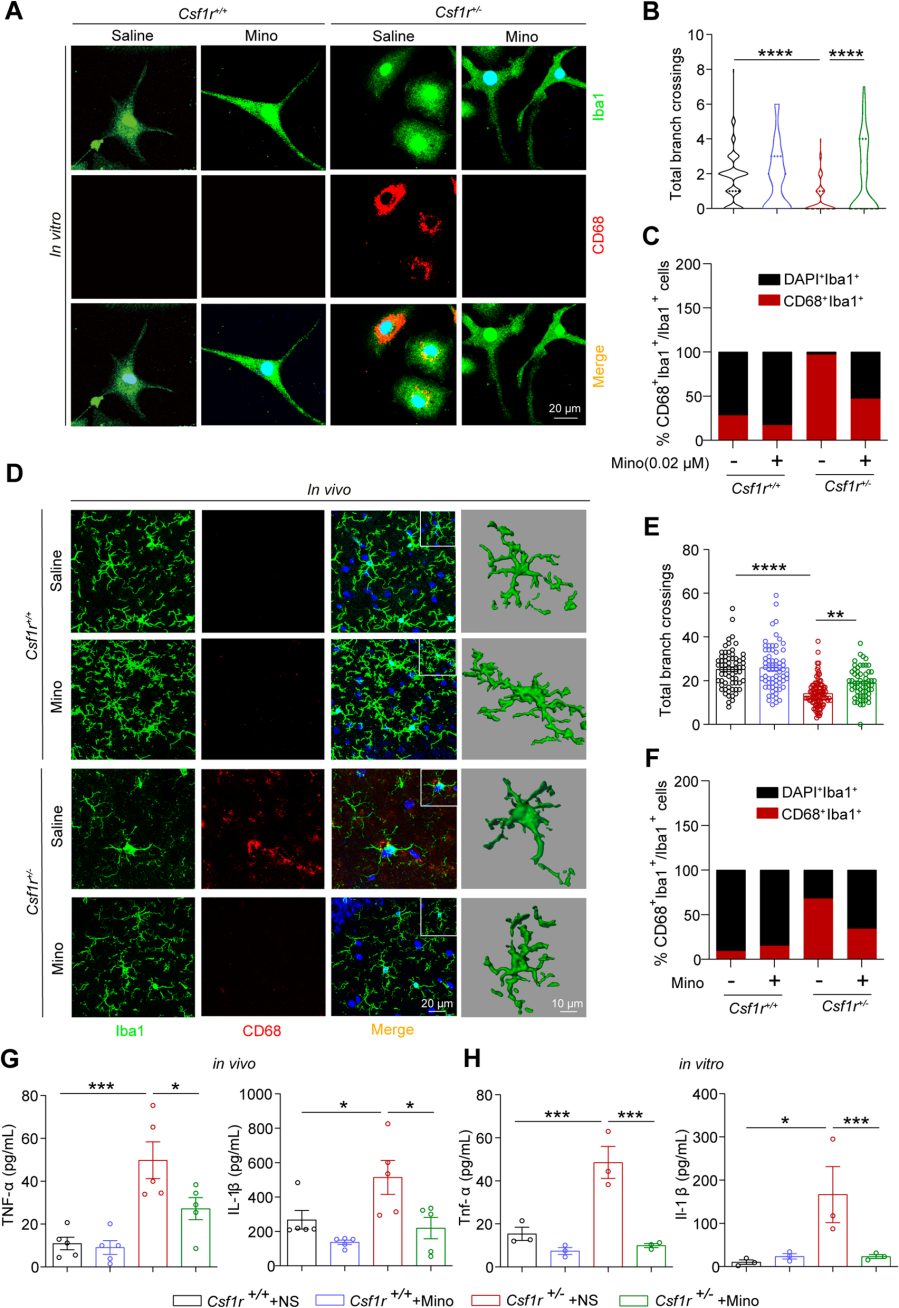
Minocycline inhibits microglial activation and ameliorates pro-inflammation in *Csf1r*^+/−^ microglia and *Csf1r*^+/−^ mouse brain. **A** *Csf1r*^+*/*+^ or *Csf1r*^+/−^ microglia were treated or not with 0.02 μM minocycline for 12 h. Representative images of Iba1^+^CD68^+^ microglia. **B** Statistical analysis of the number of microglial processes in each group (*n* = 3 independent experiments). **C** Statistical analysis of the number of Iba1^+^CD68^+^ microglia in each group (*n* = 3 independent experiments). **D** Representative images of Iba1 (green) and CD68 (red) immunostaining in brain sections from 9-month-old *Csf1r*^+*/*+^ or *Csf1r*^+/−^ mice administered with minocycline or vehicle and 3D reconstruction of the Iba1^+^CD68^+^ microglia in vivo. **E** Statistical analysis of the number of microglial processes in vivo (*n* = 5 mice per group). **F** Statistical analysis of the proportion of Iba1^+^CD68^+^ microglia to total Iba1^+^ microglia in vivo (*n* = 5 mice per group). **G** Statistical analysis of TNF-α and IL-1β protein levels in cerebral lysates of *Csf1r*^+*/*+^ or *Csf1r*^+/−^ mice treated with minocycline or vehicle (*n* = 5 mice per group). **H** Statistical analysis of TNF-α and IL-1β protein levels in conditioned medium of *Csf1r*^+*/*+^ or *Csf1r*^+/−^ primary microglia treated with minocycline or vehicle (*n* = 3 independent experiments). Data are presented as means ± SEM. *P*-values were calculated using two-way ANOVA post-Sidak’s multiple comparisons tests. *p < 0.05, **p < 0.01, ***p < 0.001, ****p < 0.0001. *Mino,* minocycline

The "microglia inhibitor", minocycline, has been reported to confer potential immunotherapeutic effects in several neurodegenerative diseases [13, 16, 32–35]. We next examined whether minocycline could effectively reverse the activation phenotypes of microglia in our model of ALSP. Indeed, minocycline exposure caused an increase in branch crossings and a significant reduction in the number of Iba1^+^CD68^+^ cells in *Csf1r*^+/−^ primary microglia compared with untreated *Csf1r*^+/−^ controls (Fig. 4A–C). Furthermore, minocycline treatment could partially inhibit microglial activation in forebrain sections of *Csf1r*^+/−^ mice, with the large majority of cells displaying a relatively resting morphology, increased arborization of the processes, and decreased Iba1^+^CD68^+^ cell populations (Fig. 4D–F). Relative expression analysis by qPCR showed that *Tnf-α* and *Il-1β* transcription was significantly decreased or in a trend of reduction in *Csf1r*^+/−^ microglia treated with minocycline compared to untreated controls, both in vivo and in vitro (Additional file 7: Fig. S6A, B). Furthermore, IL-1β and TNF-α protein levels were also significantly reduced in *Csf1r*^+/−^ mice and *Csf1r*^+/−^ microglia treated with minocycline compared with their respective *Csf1r*^+/−^ placebo-treated controls (Fig. 4G, H), which together indicated that pro-inflammatory cytokine production was reduced in response to minocycline. These results suggested that the pro-inflammatory activation of *Csf1r*^+/−^ microglia was inhibited by minocycline both in vitro and in vivo.

### Minocycline can partially rescue the behavioral deficits of *Csf1r*^+/−^ mice

**Fig. 5:**
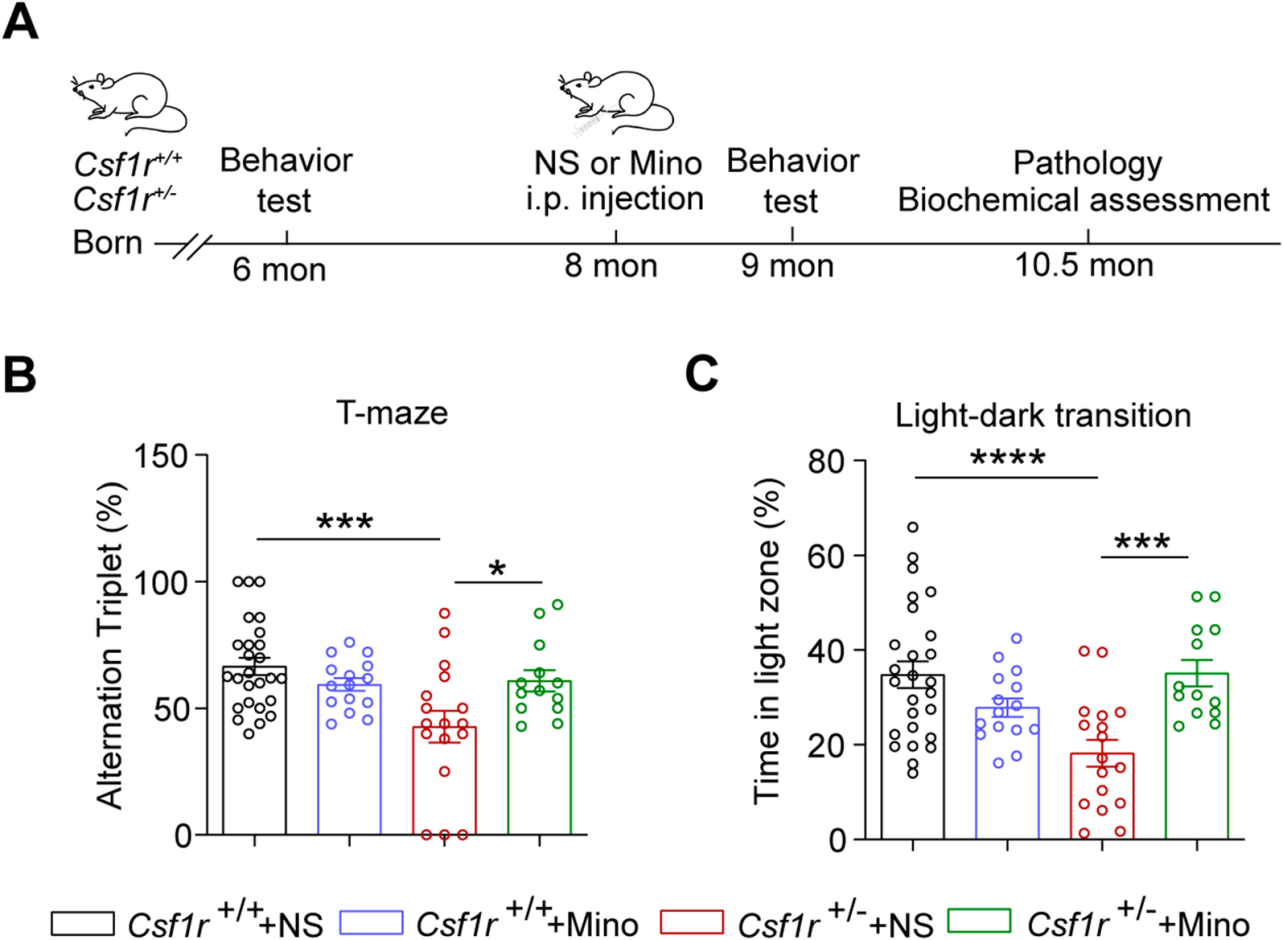
Minocycline partially improves behavioral defects in *Csf1r*^+/−^ mice**. A** Schematic timeline of the minocycline treatment timeline. Eight-month-old *Csf1r*^+*/*+^ or *Csf1r*^+/−^ mice were intraperitoneally (i.p.) administrated with 50 mg/kg minocycline or saline for one month, followed by behavioral and pathological analyses. **B** Percentage of alternation triplet time within 5 min in the T-maze test following minocycline administration (*n* = 13–26 mice). **C** Time spent in the white box in light–dark transition test following minocycline administration (*n* = 13–26 mice). Data are presented as means ± SEM. *P*-values were calculated using Two-way ANOVA post-Sidak’s multiple comparisons tests. *p < 0.05, ***p < 0.001, ****p < 0.0001. Mino, minocycline

Previous studies have provided evidence demonstrating that ALSP is a primary microgliopathy [11]. Given the abnormal microglial activation in *Csf1r*^+/−^ mice and the effective microglial inhibition by minocycline, we then sought to determine whether inhibiting microglial activation by minocycline could reverse the behavioral and pathological deficits displayed in the *Csf1r*^+/−^ mouse model of ALSP. Assessment of cognitive and emotional function in *Csf1r*^+/−^ mice by T-maze and light–dark transition test, respectively, showed that these functions were significantly restored in *Csf1r*^+/−^ treated with minocycline (Fig. 5A–C), although not to levels displayed by WT *Csf1r*^+/+^ littermates. These results suggested that minocycline could improve the behavioral deficits associated with *Csf1r*^+/−^ heterozygosity in ALSP model mice.

### Minocycline reduces demyelination and restores synaptic density in *Csf1r* haploinsufficient mouse brain

**Fig. 6:**
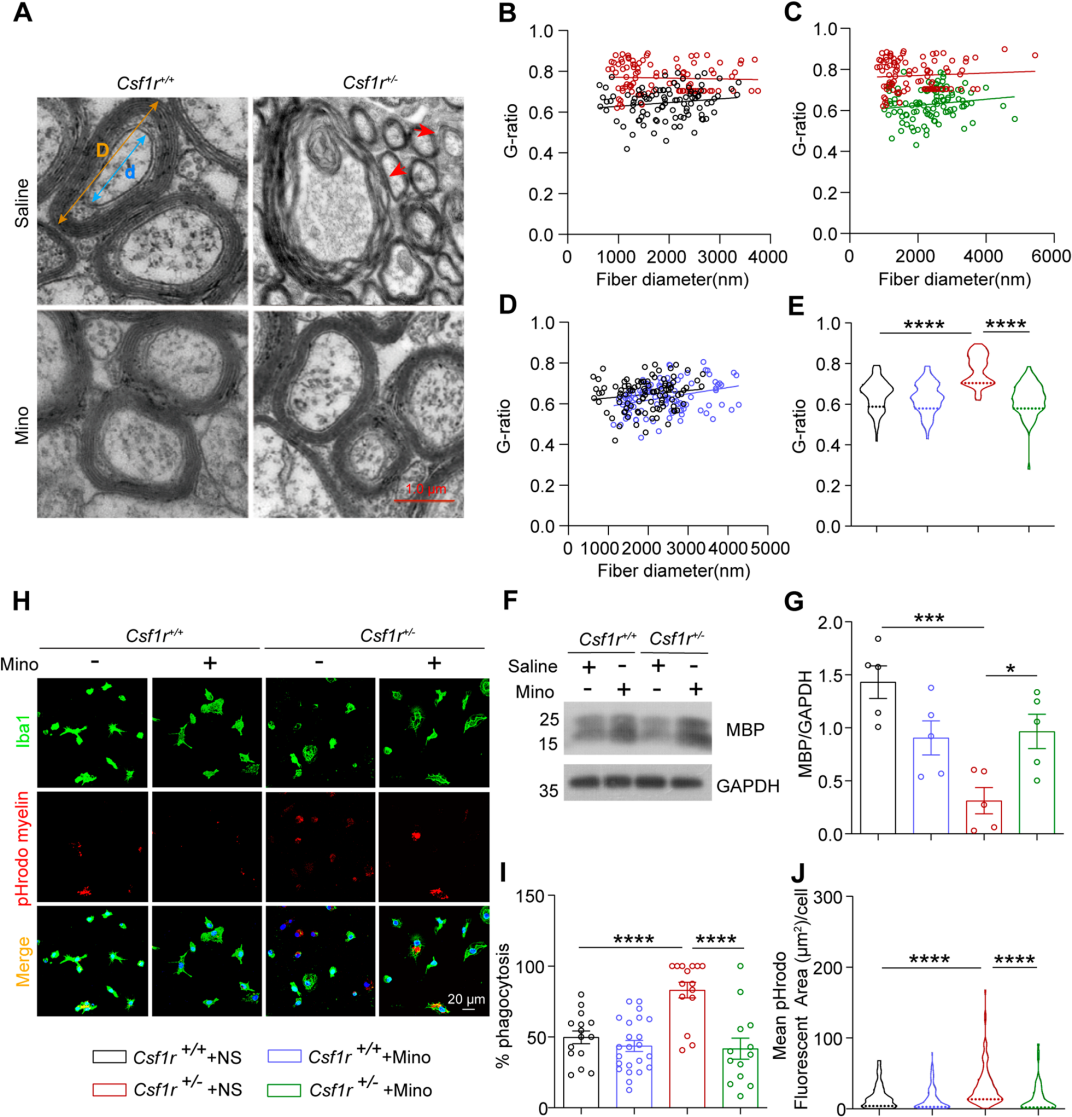
Minocycline restores the demyelination in corpus callosum by inhibiting microglial phagocytosis of myelin sheaths in *Csf1r*^+/−^ mice. **A** Electron micrographs of axons in sagittal corpus callosum sections from 9-month-old *Csf1r*^+*/*+^ or *Csf1r*^+/−^ mice administrated with minocycline or saline control. G-ratio was calculated as the ratio of the inner diameter of the myelin sheath (d) to the outer diameter of the myelin sheath (D). The red arrow indicates loosened or collapsed myelin sheaths. **B** Scatter plot showing differences in the slope of myelin sheaths between *Csf1r*^+*/*+^ and *Csf1r*^+/−^ mice. Lines indicate the regression equation (*n* = 4 mice, 96 ~ 98 axons per group). **C** Scatter plot showing differences in the slope of myelin sheaths between *Csf1r*^+/−^ mice treated with minocycline or saline vehicle (*n* = 4 mice, 98 axons per group). Lines indicate the regression equation. **D** Scatter plot showing differences in the slope of myelin sheaths between *Csf1r*^+*/*+^ mouse treated with minocycline or saline (*n* = 4 mice, 95 ~ 98 axons per group). Lines indicate the regression equation. **E** Distribution of G-ratio in *Csf1r*^+*/*+^ or *Csf1r*^+/−^ mice treated or not with minocycline (*n* = 4 mice per group). **F** Western blot detection of myelin basic protein (MBP) in the forebrain lysates from *Csf1r*^+*/*+^ or *Csf1r*^+/−^ mice treated or not with minocycline. **G** MBP protein levels quantified by densitometry ratio with GAPDH for comparison (n = 5 mice per group). **H** *Csf1r*^+*/*+^ or *Csf1r*^+/−^ microglia treated or not with 0.02 μM minocycline and co-cultured with red fluorescent dye (pHrodo)-labeled myelin sheaths for 3 h. Representative images of Iba1^+^ microglia after phagocytosis of red fluorescent dye (pHrodo)-labeled myelin sheaths. **I** Quantification of the percentage of Iba1^+^ microglia engulfing pHrodo myelin to total Iba1^+^ microglia (*n* = 3 independent experiments). **J** Quantification of microglial phagocytosis of myelin sheaths based on mean pHrodo fluorescent area in Iba1^+^ cells (*n* = 3 independent experiments). Data are presented as means ± SEM. *P*-values were calculated using Two-way ANOVA post-Sidak’s multiple comparisons tests. *p < 0.05, ***p < 0.001, ****p < 0.0001. Mino, minocycline

Callosal atrophy has been observed in brain tissue of humans with ALSP and demyelination is a hallmark of the *Csf1r* haploinsufficiency in mouse brain. We therefore investigated the effects and mechanisms by which minocycline alleviated the pathological characteristics of ALSP in *Csf1r*^+/−^ male mice. Similar to the effects on microglial activation observed above, demyelination was significantly improved in forebrain sections of minocycline-treated *Csf1r*^+/−^ mice compared to that in the saline-treated *Csf1r*^+/−^ control mice (Fig. 6A–E). Moreover, levels of the myelin marker, MBP, were higher in forebrain samples of *Csf1r*^+/−^ mice treated with minocycline compared to that in the saline control group (Fig. 6F–G). Further examination of phagocytic capacity as a potential mechanism contributing to the therapeutic effects of minocycline indicated that microglial engulfment of myelin sheaths was lower in primary *Csf1r*^+/−^ microglia treated with minocycline than that in the *Csf1r*^+/−^ saline-treated control cells (Fig. 6H–J).

**Fig. 7:**
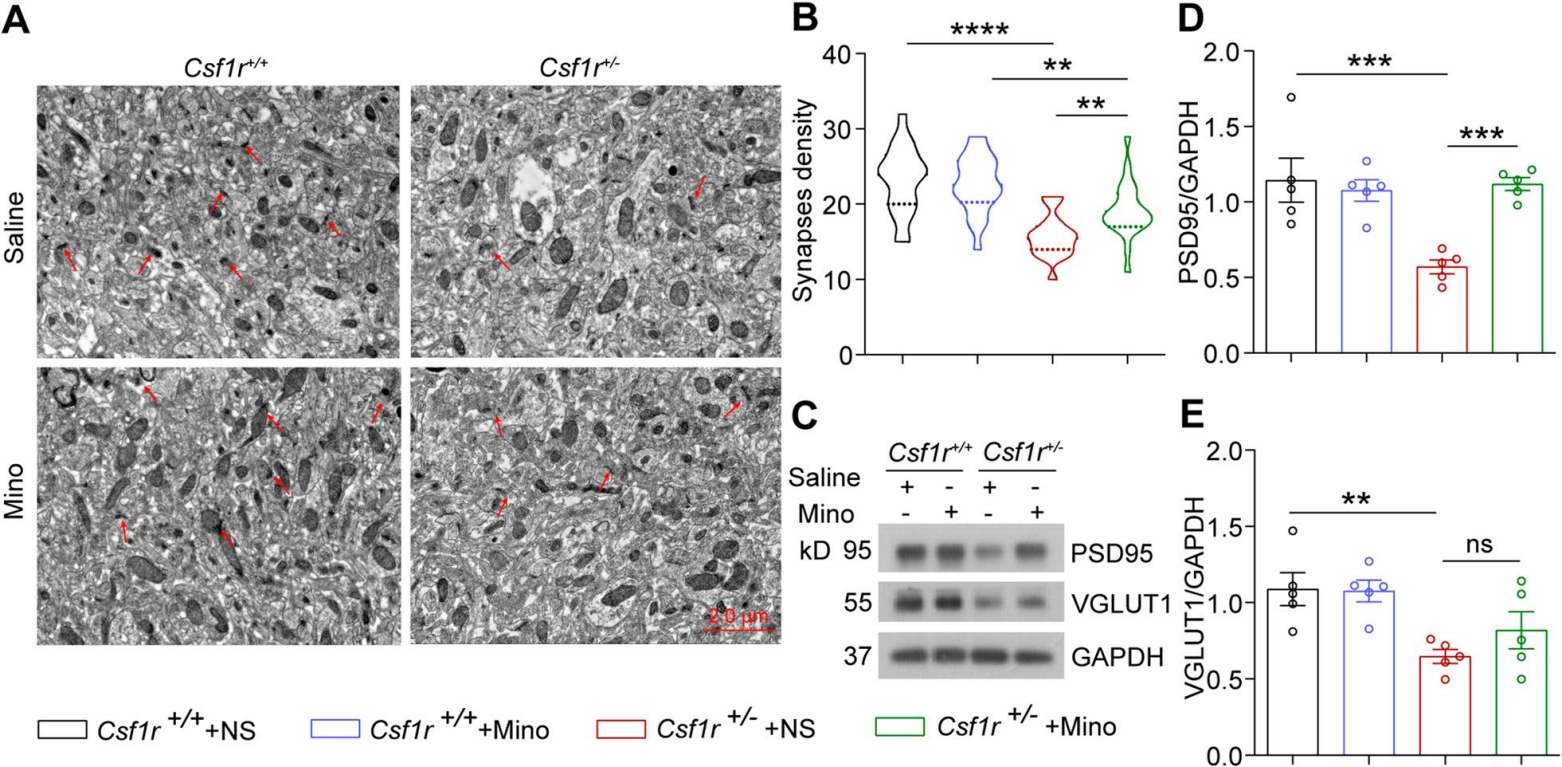
Minocycline partially restores synaptic density in forebrain tissue of *Csf1r*^+/−^ mice. **A** Electron micrographs of synapse density in the CA1 area of forebrain sections from 9-month-old *Csf1r*^+*/*+^ or *Csf1r*^+/−^ mice administered or not minocycline. **B** The number of synapses in the CA1 area of *Csf1r*^+*/*+^ or *Csf1r*^+/−^ mice treated or not with minocycline was analyzed (*n* = 4 mice per group). **C** Western blot detection of postsynaptic membrane protein marker (PSD95) and presynaptic membrane protein marker (VGLUT1) in forebrain lysates of *Csf1r*^+*/*+^ or *Csf1r*^+/−^ mice treated or not with minocycline. **D** PSD95 protein levels quantified by densitometry with GAPDH for comparison (*n* = 5 mice per group). **E** VGLUT1 protein levels quantified by densitometry with GAPDH for comparison (*n* = 5 mice per group). Data are presented as means ± SEM. *P*-values were calculated using two-way ANOVA post-Sidak’s multiple comparisons tests. **p < 0.01, ***p < 0.001, ****p < 0.0001, ns, no significance. Mino, minocycline

Previous reports indicate that minocycline also affects microglial engulfment of synapses [36]. We found that synapse density was significantly lower in male mice *Csf1r* haploinsufficiency compared to that in their WT littermates, which could be partially rescued by minocycline administration in *Csf1r*^+/−^ male mice in vivo (Fig. 7A, B). Furthermore, synaptic protein levels of PSD95 (postsynaptic protein) and VGLUT1 (presynaptic protein), which were decreased in forebrain sections of *Csf1r*^+/−^ mice, were restored (though not significantly in VGLUT1) in response to minocycline, albeit at still lower levels than that in WT littermates (Fig. 7C–E), which was consistent with our observations of improved synapse density. These cumulative results suggested that minocycline could improve the impaired pathological phenotype of *Csf1r*^+/−^ mouse brain.

### Minocycline regulates cellular metabolism and organization in brains of *Csf1r* haploinsufficient mice

**Fig. 8:**
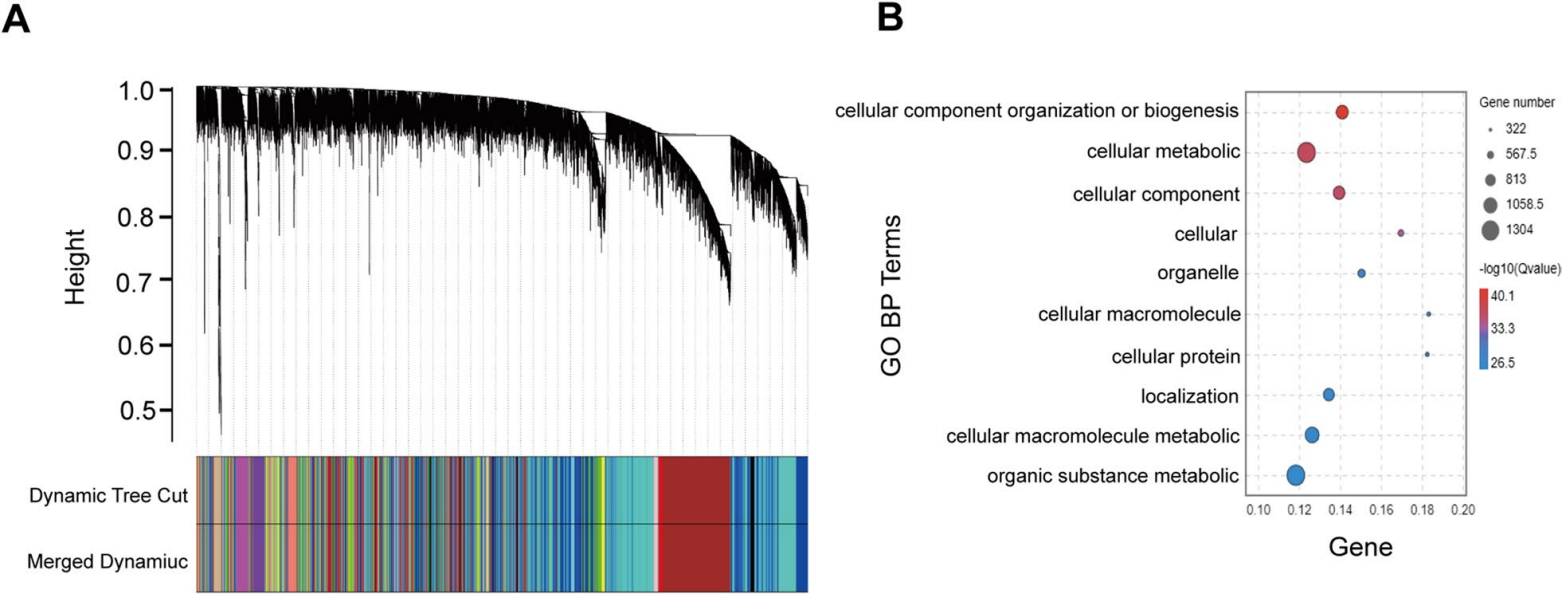
WGCNA in the forebrain of *Csf1r*^+/−^ mice treated or not with minocycline.** A** Dendrogram of hierarchical clustering analysis of all differentially expressed genes (DEGs) comparing forebrains of 9-month-old *Csf1r*^+*/*+^ or *Csf1r*^+/−^ mice treated or not with minocycline (*n* = 4 mice per group). Band color shows gene modules obtained by automatic single-block analysis. **B** GO analysis of DEGs in the module correlated with *Csf1r*^+/−^ response to minocycline treatment (brown) in the WGCNA dendrogram. The top ten high confidence GO biological process terms are shown. Mino, minocycline

To further examine the possible mechanisms by which minocycline exposure led to beneficial therapeutic effects on behavioral and pathological deficits of *Csf1r*^+/−^ ALSP model mice, we collected cerebral samples from *Csf1r*^+/+^ or *Csf1r*^+/−^ male mice treated or not with minocycline for RNA-seq analysis. Weighted gene co-expression network analysis (WGCNA) identified a set of core modules and central genes associated with minocycline treatment, and hierarchical clustering and with dynamic tree clipping (Fig. 8A) identified a single module (brown) that was highly correlated with changes in pathological severity following minocycline treatment. GO enrichment analysis exploring the functional annotation of genes in this module intriguingly showed enrichment in biological processes associated with ‘cellular metabolic process’ and ‘cellular component organization or localization’ (Fig. 8B; see Additional file 6: Table S6 for a complete list of gene clusters), suggesting a potentially protective role for minocycline in mitigating the pathological ALSP phenotype via regulation of cellular metabolism and cellular organization in microglia.

## Discussion

A recent study has suggested that CSF1R heterozygosity in microglia is sufficient to induce pathogenesis in ALSP [11]. CSF1R-related leukoencephalopathy is therefore considered a primary microgliopathy of the central nervous system (CNS). The effects of losing one *Csf1r* allele or mutation of *Csf1r* have been thoroughly documented in previous studies using similar experimental approaches in mouse, rat, or zebrafish models. However, the underlying molecular mechanisms by which CSF1R deficiency leads to neurodegeneration has remained unclear and effective therapeutic strategies are still lacking in clinic.

**Fig. 9:**
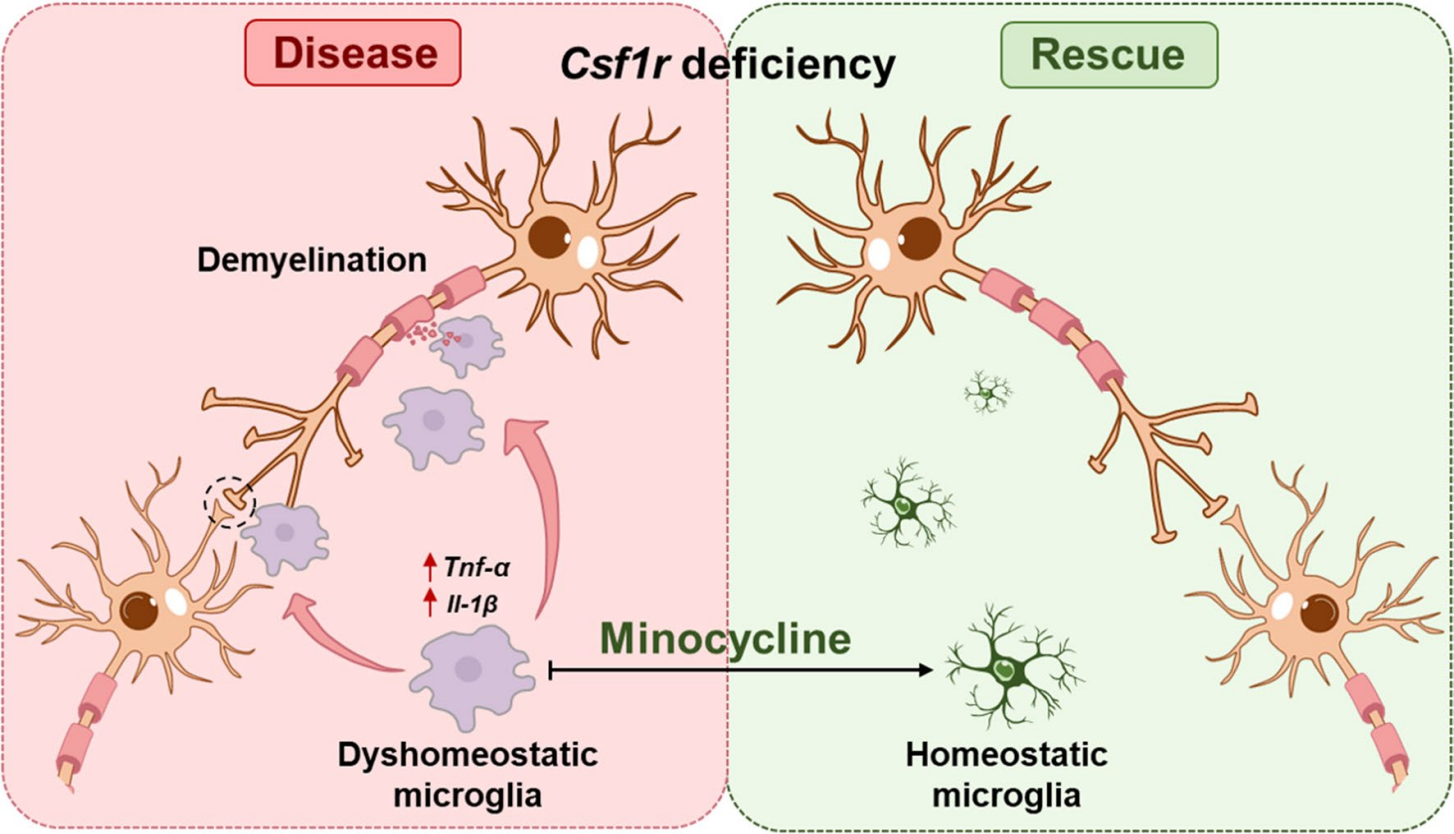
Schematic of the mechanism of minocycline action alleviating ALSP pathology in *Csf1r*^+/−^ mice. *Csf1r* haploinsufficiency results in the dyshomeostasis of microglia, including an enhanced inflammatory and myelin-phagocytic phenotype, a primary cause of ALSP pathogenesis. Minocycline significantly improves ALSP pathogenesis through inhibition of inflammation and myelin phagocytosis by microglia

In this study, we demonstrated that CSF1R deficiency results in abnormal microglial activation, which is critical for the pathogenesis of ALSP. This finding is consistent with recent studies in human post-mortem brain tissues [37] and in *Csf1r*^+/−^ mice [11, 38] that showed CSF1R-dependent leukoencephalopathy is mediated by the activation of microglia. Importantly, findings in this study indicate that modulating microglial activation with the microglial inhibitor, minocycline, can partially restore the behavioral and pathological deficits characteristic of *Csf1r*^+/−^ ALSP mice (Fig. 9).

Intriguingly, several studies have reported finding microglial dyshomeostasis in human ALSP patients [37, 39]. For instance, one study showed that increased CD68 immunoreactivity mainly occurs in the superficial cerebral white matter of HDLS patients [37]. Another study revealed that microglia in the cerebral white matter have a dyshomeostatic phenotype in HDLS [37]. Additionally, observations of increased microglial activation has been reported in brain samples of adult *Csf1r*^+/−^ and *Csf1r*^E631K/+^ mice [40], as well as CSF1R-mutant human brains [10, 29, 41, 42]. Consistent with these reports, we found that *Csf1r* haploinsufficient microglia have increased proportions of Iba1^+^CD68^+^ cells with dramatically activated morphological features in our ALSP mouse model. Furthermore, microgliosis has also been observed in *Csf1r*^+/−^ mice and mice with microglial depletion (M*Csf1r*^het^) [10, 11, 38]. By contrast, heterozygous *Csf1r* knockout rats had no detectable pathological brain phenotype nor elevated microglial densities [43], which may be a premature conclusion stemming from a small sample size (*n* = 5) of *Csf1r*^+/−^ rats [29]. However, decreased microglial density has also been reported in adult mice with inducible *Csf1r*^+/−^ heterozygosity [12], *Csf1r*^E631K/+^ mice [40], and *Csf1r* mutant zebrafish [44]. Multiple studies have shown that microglial abundance is reduced in *Csf1r* haploinsufficient animal models and CSF1R-associated ALSP patients [10, 30, 43]. Consistent with these previous studies, immunofluorescent staining for Iba1 in the current study confirmed that microglia density is indeed decreased in the cortex and hippocampus of *Csf1r*^+/−^ mice (Additional file 7: Fig. S7). Collectively, these studies suggest that microglial dyshomeostasis induced by loss of one *Csf1r* allele can serve as a key driver of ALSP pathogenesis. Nevertheless, the molecular mechanisms responsible for the abnormal *Csf1r*^+/−^ microglial morphology and function require further verification.

Notably, in this study, *Csf1r* haploinsufficient microglia also showed functional changes, including enhanced production of pro-inflammatory factors and enhanced phagocytosis of myelin sheaths, whereas other studies have reported that *Csf1r*^+/−^ microglia show an oxidative rather than not pro-inflammatory phenotype [38]. GSEA analysis showed noteworthy enrichment for genes related to the phagosome pathway and toll-like receptor pathway in adult *Csf1r*^+/−^ brains, further supporting the increased phagocytosis of myelin in ALSP. Nevertheless, these apparent disparities among observations of an inflammatory microglial state associated with CSF1R deficiency indicate the need for further investigation. Deletion of the *fms*-intronic regulatory element (FIRE), a super-enhancer in the second intron of *Csf1r*, leads to complete loss of brain microglia and resident tissue macrophages [45]. However, the FIRE mutation has no impact on the morphology or phagocytosis of *Csf1r*^ΔFIRE/ΔFIRE^ RAW 264.7 macrophage cells [45]. By contrast, CSF1R haploinsufficiency has been shown to decrease the phagocytosis capacity of beads or amyloid-β in peripheral macrophages, but not microglia, isolated from *Csf1r*^flx/wt^; *Cx3cr1*^Cre/+^ mice [46]. Our study shows that primary *Csf1r*^+/−^ microglia displayed no obvious changes in phagocytic capacity in response to beads, but exhibit enhanced phagocytosis in response to myelin sheaths, suggesting that *Csf1r*^+/−^ leads to microglia-specific changes in phagocytic capacity. We have previously reported that expression of *Trem2*, a crucial gene for cell movement and phagocytosis, is significantly increased in *Csf1r* knockdown microglia [19]. Based on patterns of gene expression observed in the *Csf1r*^+/−^ mouse brain, it is possible that C1q, a crucial phagocytic signal in synapses, may be responsible for the enhanced myelin engulfment. Additionally, our recent findings suggest that CSF1R interacts with TREM2 to synergistically regulate microglial survival, while TREM2 modulates microglial migration and phagocytosis via the FAK/Cdc42 signaling pathway [47]. Another recent study reported that activated microglia can drive demyelination directly via CSF1R signaling [48]. Thus, further research is warranted to determine whether TREM2 or C1q are also involved in the phagocytic capacity of *Csf1r*^+/−^ microglia.

Abnormal astrocytic phenotypes and astrocytic endocytosis, which are sufficient to cause leukodystrophy, have been also observed in *Csf1ra*^*V614M/*+^ zebrafish [44]. Another study showed that GFAP immunoreactivity was reduced in 7-week-old *Csf1r*^E631K/+^ mice but was not altered in 43-week-old *Csf1r*^E631K/+^ mice [40]. However, it should be noted that immunofluorescent staining of GFAP showed increased levels in brain sections of 9-month-old *Csf1r*^+/−^ mice in this study (Additional file 7: Fig. S8). Therefore, we cannot exclude the possibility that astrocytes may also contribute to the pathogenesis of ALSP.

Although microglia replacement therapies have emerged as potentially effective modalities for treating ALSP, clinical trials are still needed [49, 50]. The prominent activation of *Csf1r*^+/−^ microglia in the brain prompted our investigation of microglial inhibition as a possible approach to reversing ALSP pathogenesis. Accumulating studies have confirmed that minocycline, a second-generation tetracycline antibiotic, can inhibit microglial activation in patients and animal models [20, 34, 35, 51–53]. Minocycline is lipophilic and can easily pass through the blood–brain barrier. However, in this study, even after minocycline treatment, the number of processes and the expression of CD68 were not restored to levels comparable with WT control animals, although improved from that in untreated controls. This finding might be related to incomplete transport of the drug through the blood–brain barrier. Hypometabolism detected by [^18^F]-fluorodeoxyglucose PET has been reported in frontoparietal regions of ALSP patients [1, 3, 54]. In this study, ‘cellular metabolic process’ and ‘cellular component organization or localization’ were both enriched pathways in the minocycline treatment group. Thus, further investigation of the specific cellular metabolic process involved in the pathological progress of ALSP is warranted in future studies.

These collective studies indicate that microglial activation is a primary cause of CSF1R-associated leukoencephalopathy and inhibiting microglial activation by minocycline may be an effective strategy to improve behavioral performance, based on our findings in our mouse model of ALSP. Our study also reveals myelin engulfment and inflammatory response as two molecular mechanisms through which CSF1R deficiency-mediated microglial activation leads to ALSP-associated pathologies. This study thus helps unravel the pathological function of CSF1R deficiency in ALSP and provides therapeutic targets for alleviating microglial dysfunction associated with leukoencephalopathy.

## Conclusions

This study provides the first report of which we are aware showing that *Csf1r* haploinsufficiency results in early impaired synaptic plasticity, as evaluated by LTP assay and demonstrates the aberrant activation of microglia in *Csf1r*^+/−^ mice and *Csf1r*^+/−^ primary microglia. Notably, this study also provides a strong therapeutic basis for developing minocycline as a modulator of microglial homeostasis for patients with CSF1R variants at high risk of ALSP pathogenesis.

## Supplementary Information

**Additional file 1: Table S1.** The detailed informationof mice used in each behavioral experiment before or after minocycline treatment.

**Additional file 2:** **Table S2. **Primers for the target genes of qRT-PCR.

**Additional file 3.** Differentially expressed genes (DEGs) from *Csf1r*^+/+^ and *Csf1r*^+/—^ mice.

**Additional file 4.** Enrichment genes in phagosome pathway from *Csf1r*^+/+^ and *Csf1r*^+/—^ mice.

**Additional file 5.** Enrichment genes in toll-like receptor pathway from *Csf1r*^+/+^ and *Csf1r*^+/—^ mice.

**Additional file 6.** Genes in brown module from *Csf1r*^+/+^ and *Csf1r*^+/—^ mice associated with minocycline treatment.

**Additional file 7: Figure S1.** Identification of CSF1R expression in* Csf1r*^*+/—*^ microglia and *Csf1r*^+/−^ mouse brain. **Figure S2.** CSF1R haploinsufficiency does not alter the synaptic function in female mouse brain. **Figure S3.** CSF1R haploinsufficiency results in noteworthy changes in the enrichment gene sets of phagosome or toll-like receptor pathway. **Figure S4.** CSF1R haploinsufficiency results in enhancement in the mRNA levels of *Tnf-α* and *Il-1β *both in vivo and in vitro. **Figure S5.** CSF1R haploinsufficiency does not affect the phagocytosis of microsphere beads by microglia. **Figure S6.** Minocycline exposure partially inhibits the mRNA levels of *Tnf-α* and *Il-1β *in *Csf1r*^*+/—*^ microglia or *Csf1r*^*+/—*^ mouse brain. **Figure S7.** The density of microglia is reduced in *Csf1r*^*+/—*^ mouse brain. **Figure S8.** Astrocyte is activated in *Csf1r*^*+/—*^ mouse brain.

## Data Availability

All datasets generated during the current study are available from the corresponding author on reasonable request.

## Abbreviations

ALSP: Adult-onset leukoencephalopathy with axonal spheroids and pigmented glia
CSF1R: Colony-stimulating factor 1 receptor
LTP: Long-term potentiation
POLD: Pigmented orthochromatic leukodystrophy
HDLS: Hereditary diffuse leukoencephalopathy with axonal spheroids
AD: Alzheimer’s disease
ALS: Amyotrophic lateral sclerosis
PD: Parkinson’s disease
FTD: Frontal temporal dementia
TEM: Transmission electron microscopy
