# Supplementary material for: Minocycline protects against microgliopathy in a Csf1r haplo-insufficient mouse model of adult-onset leukoencephalopathy with axonal spheroids and pigmented glia (ALSP)
Source: J Neuroinflammation. 2023 May 31;20:134. doi: 10.1186/s12974-023-02774-1 (PMC10234026; doi:10.1186/s12974-023-02774-1)
Supplement: Supplementary file 1 — Additional file 1: Table S1. The detailed informationof mice used in each behavioral experiment before or after minocycline treatment. [file 12974_2023_2774_MOESM1_ESM.docx]

**Additional file 1: Table S1. The detailed information (number, sex, and age) of mice used in each behavioral experiment before or after minocycline treatment.**

|  |  |  | **Male** | | **Female** | |  |  |  |
| --- | --- | --- | --- | --- | --- | --- | --- | --- | --- |
|  | **Subject** |  | ***Csf1r*^+/+^** | ***Csf1r*^+/-^** | ***Csf1r*^+/+^** | ***Csf1r*^+/-^** | **Total** | **Age at Start** | **Age at End** |
| **Pre-treatment** | **Learning and memory ability** | **T-maze** | 15 | 14 | 15 | 14 | 58 | 6 mon | 7.5 mon |
|  |  | **Novel object recognition test** | 12 | 15 | 8 | 13 | 48 | 6 mon | 7.5 mon |
|  | **Anxiety-like behavior** | **Open field test** | 15 | 15 | 15 | 13 | 58 | 6 mon | 7.5 mon |
|  |  | **Light-dark transition test** | 15 | 15 | 15 | 14 | 59 | 6 mon | 7.5 mon |
|  | **Depression-like behavior** | **Sucrose preference test** | 14 | 15 | 14 | 12 | 55 | 6 mon | 7.5 mon |
|  | **Subject** |  | ***Csf1r*^+/+^ + NS** | ***Csf1r*^+/+^ + mino** | ***Csf1r*^+/-^**  **+ NS** | ***Csf1r*^+/-^**  **+ mino** |  |  |  |
| **Post-treatment** | **Learning and memory ability** | **T-maze test** | 26 | 15 | 17 | 13 | 71 | 9 mon | 10.5 mon |
|  | **Anxiety-like behavior** | **Light-dark transition**  **test** | 26 | 15 | 17 | 13 | 71 | 9 mon | 10.5 mon |

In pre-treatment behavioral tests, one mouse in the *Csf1r*^+/-^ group did not move throughout the T-maze experiment and no movement trajectory was recorded for this individual. Individuals that had extreme positional preferences during the old object recognition training were excluded from the test. One mouse in the *Csf1r*^+/-^ group died during the open-field test. One mouse in the *Csf1r*^+/+^ group was excluded due to the leakage of sucrose water bottle and three mice in the *Csf1r*^+/-^ group died during the sucrose preference test.
