# Supplementary material for: Minocycline protects against microgliopathy in a Csf1r haplo-insufficient mouse model of adult-onset leukoencephalopathy with axonal spheroids and pigmented glia (ALSP)
Source: J Neuroinflammation. 2023 May 31;20:134. doi: 10.1186/s12974-023-02774-1 (PMC10234026; doi:10.1186/s12974-023-02774-1)
Supplement: Supplementary file 2 — Additional file 2: Table S2. Primers for the target genes of qRT-PCR. [file 12974_2023_2774_MOESM2_ESM.docx]

**Additional file 2: Table S2. Primers for the target genes of qRT-PCR.**

| Name | GenBank | Primer sequences (Forward/Reverse) |
| --- | --- | --- |
| *Actb* | NM_11461 | TCTTGGGTATGGAATCCTGTGGCA  TCTCCTTCTGCATCCTGTCAGCAA |
| *Csf1r* | NM_12978 | GGTTGTAGAGCGGGTGAAA  AAGAGTGGGCCGATCTTTG |
| *Il-1β* | NM_16176 | GCACACCCACCCTGCA  ACCGCTTTTCCATCTTCTTCTT |
| *Tnf-α* | NM_21926 | CTCCAGGCGGTGCCTATG  GGGCCATAGAACTGATGAGAGG |
