# Supplementary material for: Minocycline protects against microgliopathy in a Csf1r haplo-insufficient mouse model of adult-onset leukoencephalopathy with axonal spheroids and pigmented glia (ALSP)
Source: J Neuroinflammation. 2023 May 31;20:134. doi: 10.1186/s12974-023-02774-1 (PMC10234026; doi:10.1186/s12974-023-02774-1)
Supplement: Supplementary file 7 — Additional file 7: Figure S1. Identification of CSF1R expression in Csf1r+/— microglia and Csf1r+/− mouse brain. Figure S2. CSF1R haploinsufficiency does not alter the synaptic function in female mouse brain. Figure S3. CSF1R haploinsufficiency results in noteworthy changes in the enrichment gene sets of phagosome or toll-like receptor pathway. Figure S4. CSF1R haploinsufficiency results in enhancement in the mRNA levels of Tnf-α and Il-1β both in vivo and in vitro. Figure S5. CSF1R haploinsufficiency does not affect the phagocytosis of microsphere beads by microglia. Figure S6. Minocycline exposure partially inhibits the mRNA levels of Tnf-α and Il-1β in Csf1r+/— microglia or Csf1r+/— mouse brain. Figure S7. The density of microglia is reduced in Csf1r+/— mouse brain. Figure S8. Astrocyte is activated in Csf1r+/— mouse brain. [file 12974_2023_2774_MOESM7_ESM.zip › New folder/Supplementary Figure Legends.docx]

**Additional file 1: Figure Legends**

**Figure S1.** **Identification of CSF1R expression in *Csf1r^+/-^* microglia and *Csf1r*^+/-^ mouse brain.**

1. Schematic diagram of *Csf1r^+/-^* mouse model constructed by CRISPR/Cas9 genome editing. **B.** Genotyping of *Csf1r*^+/+^, *Csf1r*^+/-^ and *Csf1r*^-/-^ mice by PCR. WT: 116 bp, KO: 94 bp. **C.** Representative images of Western blot showing an allele-dependent expression of CSF1R in *Csf1r^+/+^*, *Csf1r^+/-^* and *Csf1r^-/-^* mouse brain at postnatal 0-3 days. **D.** CSF1R protein levels in mouse brain were quantified by densitometry ratio to α-tubulin for comparison (n=3 mice per group). **E.** *Csf1r* mRNA levels in mouse brain were quantified by quantitative RT-PCR (n=3 mice per group). **F.** Representative images of Western blot showing allele-dependent alteration of CSF1R in *Csf1r^+/+^*, *Csf1r^+/-^* and *Csf1r^-/-^* primary microglia. **G.** CSF1R protein levels in primary microglia were quantified by densitometry ratio to α-tubulin for comparison (n = 3 independent experiments). **H.** *Csf1r* mRNA levels were in primary microglia were quantified by quantitative RT-PCR (n = 3 independent experiments). Data are presented as means ± SEM. *P*-values were calculated using One-way ANOVA. **p*<0.05, ***p*<0.01, ****p*<0.001, *****p*<0.0001.

**Figure S2.** **CSF1R haplo-insufficiency does not alter the synaptic function in female mouse brain.**

**A.** Time series plots of evoked field potentials were recorded in the CA1 area of hippocampal slices from 8- or 9-month-old *Csf1r*^+/+^ or *Csf1r*^+/-^ female mice. HFS, high-frequency stimulation. **B.** The average fEPSP amplitude over the last 10 min of LTP recording was quantified. *Csf1r*^+/+^ (n = 3 mice, 9 slices), *Csf1r*^+/-^ (n = 4 mice, 10 slices). Data are presented as means ± SEM. *P*-values were calculated using unpaired two-tailed Student’s *t*-test. ns, no significance.

**Figure** **S3.** **CSF1R haplo-insufficiency results in noteworthy changes in** **the enrichment gene sets of phagosome or** **toll-like receptor pathway.**

**A.** Heatmap of the enrichment gene sets associated with phagosome pathway in forebrains from *Csf1r^+/+^* or *Csf1r^+/-^* mice. **B.** Heatmap of the enrichment gene sets associated with toll-like receptor pathway in forebrains from *Csf1r^+/+^* or *Csf1r^+/-^* mice. The colour key (from blue to red) of the Z-score value (−2 – 2) indicates low to high expression levels.

**Figure** **S4.** **CSF1R haplo-insufficiency results in enhancement in the mRNA levels of *Tnf-α* and *Il-1β*** **both *in vivo* and *in vitro*.**

**A.** *Tnf-α* and *Il-1β* mRNA levels in 9-month-old *Csf1r^+/+^* or *Csf1r^+/-^* mouse brain were quantified by quantitative RT-PCR (n = 5 mice per group). **B.** *Tnf-α* and *Il-1β* mRNA levels in primary *Csf1r^+/+^* or *Csf1r^+/-^* microglia were quantified by quantitative RT-PCR (n = 3 independent experiments). Data are presented as means ± SEM. *P*-values were calculated using unpaired two-tailed Student’s *t*-test. **p*< 0.05; ****p*< 0.001; *****p*< 0.0001; ns, no significance.

**Figure** **S5.** **CSF1R haplo-insufficiency does not affect the phagocytosis of microsphere beads by microglia.**

**A.** Primary cultured *Csf1r^+/+^* or *Csf1r^+/-^* microglia were treated with microsphere beads for 1 hr. Representative images of Iba1^+^ cells (green) colocalized with Beads (red) were shown. **B.** Phagocytosis of microsphere beads by microglia was quantified by the mean red fluorescent intensity in Iba1^+^ cells (n = 3 independent experiments). Data are presented as means ± SEM. *P*-values were calculated using unpaired two-tailed Student’s *t*-test. ns, no significance.

**Figure** **S6.** **Minocycline exposure partially inhibits the mRNA levels of *Tnf-α* and *Il-1β*** **in *Csf1r^+/-^* microglia or *Csf1r^+/-^* mouse brain.**

**A.** *Tnf-α* and *Il-1β* mRNA levels in 9-month-old *Csf1r^+/+^* or *Csf1r^+/-^* mouse brain treated with or not minocycline were quantified by quantitative RT-PCR (n = 5 mice per group). **B.** *Tnf-α* and *Il-1β* mRNA levels in primary *Csf1r^+/+^* or *Csf1r^+/-^* microglia treated with or not minocycline were quantified by quantitative RT-PCR (n = 3 independent experiments). Data are presented as means ± SEM. *P*-values were calculated using Two-way ANOVA post Sidak’s multiple comparisons test. **p*< 0.05; ***p*< 0.01; *****p*< 0.0001; ns, no significance.

**Figure** **S7.** **The density of microglia is reduced in *Csf1r^+/-^* mouse brain.**

**A.** Representative images of Iba1 (green) immunostaining in brain sections from 9-month-old *Csf1r^+/+^* or *Csf1r^+/-^* mice. **B.** Microglial cell number in the cortex from *Csf1r^+/+^* or *Csf1r^+/-^* mice was calculated by counting the number of Iba1^+^ cells co-localized with DAPI (n = 5 mice per group). **C.** Microglial cell number in the hippocampus from *Csf1r^+/+^* or *Csf1r^+/-^* mice was calculated by counting the number of Iba1^+^ cells co-localized with DAPI (n = 5 mice per group). Data are presented as means ± SEM. *P*-values were calculated using unpaired two-tailed Student’s *t*-test. ***p*< 0.01; *****p*< 0.0001.

**Figure** **S8.** **Astrocyte is activated in *Csf1r^+/-^* mouse brain.**

**A.** Representative images of GFAP (green) immunostaining in brain sections from 9-month-old *Csf1r^+/+^* or *Csf1r^+/-^* mice. **B.** Astrocyte cell number in the hippocampus from *Csf1r^+/+^* or *Csf1r^+/-^* mice was calculated by counting the number of GFAP^+^ cells co-localized with DAPI (n = 5 mice per group). **C.** The mean GFAP fluorescent intensity in the hippocampus from *Csf1r^+/+^* or *Csf1r^+/-^* mice was quantified by densitometry (n = 5 mice per group). Data are presented as means ± SEM. *P*-values were calculated using unpaired two-tailed Student’s *t*-test. *****p*< 0.0001; ns, no significance.
