# Supplementary figures and images for: Minocycline protects against microgliopathy in a Csf1r haplo-insufficient mouse model of adult-onset leukoencephalopathy with axonal spheroids and pigmented glia (ALSP)
Source: J Neuroinflammation. 2023 May 31;20:134. doi: 10.1186/s12974-023-02774-1 (PMC10234026; doi:10.1186/s12974-023-02774-1)

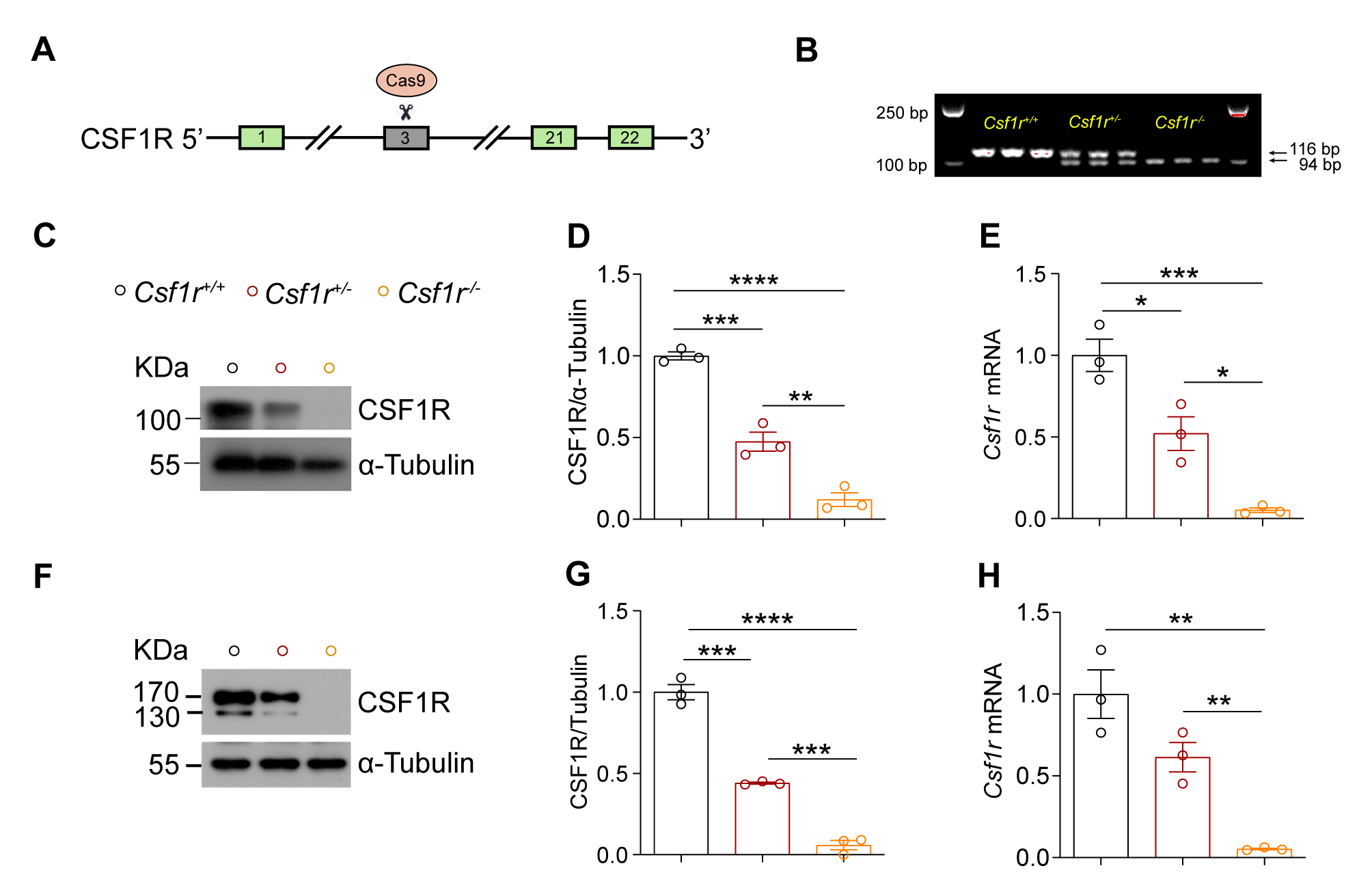

Supplement: Supplementary file 7 — Additional file 7: Figure S1. Identification of CSF1R expression in Csf1r+/— microglia and Csf1r+/− mouse brain. Figure S2. CSF1R haploinsufficiency does not alter the synaptic function in female mouse brain. Figure S3. CSF1R haploinsufficiency results in noteworthy changes in the enrichment gene sets of phagosome or toll-like receptor pathway. Figure S4. CSF1R haploinsufficiency results in enhancement in the mRNA levels of Tnf-α and Il-1β both in vivo and in vitro. Figure S5. CSF1R haploinsufficiency does not affect the phagocytosis of microsphere beads by microglia. Figure S6. Minocycline exposure partially inhibits the mRNA levels of Tnf-α and Il-1β in Csf1r+/— microglia or Csf1r+/— mouse brain. Figure S7. The density of microglia is reduced in Csf1r+/— mouse brain. Figure S8. Astrocyte is activated in Csf1r+/— mouse brain. [file 12974_2023_2774_MOESM7_ESM.zip › New folder/Supplementary Figure 1.tif]

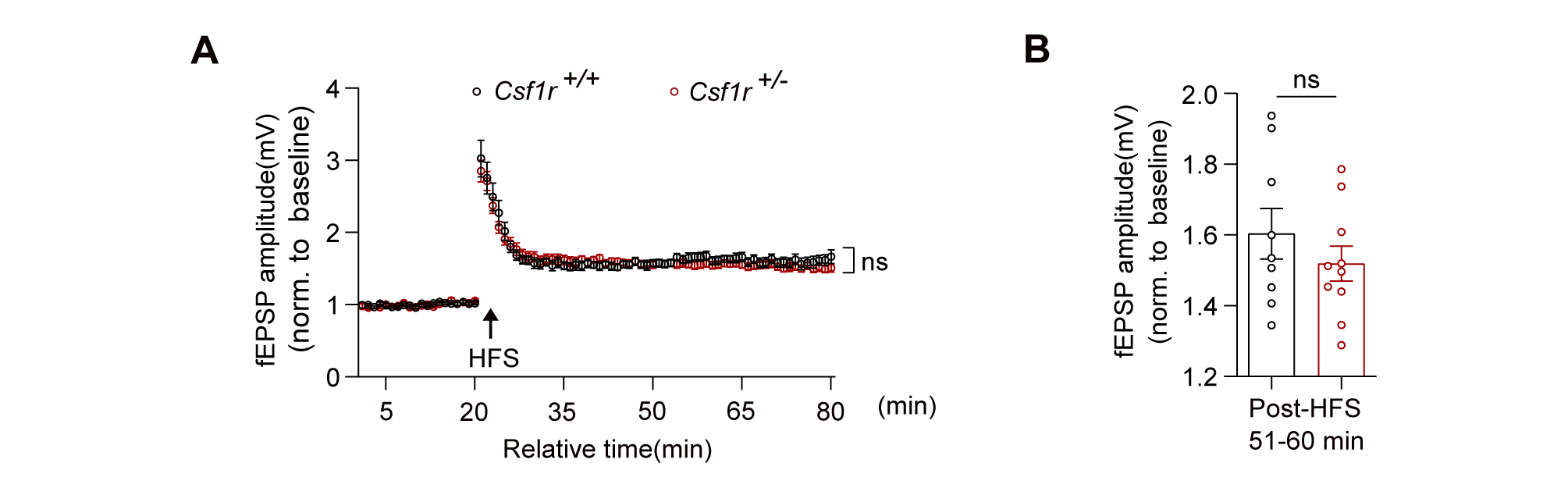

Supplement: Supplementary file 7 — Additional file 7: Figure S1. Identification of CSF1R expression in Csf1r+/— microglia and Csf1r+/− mouse brain. Figure S2. CSF1R haploinsufficiency does not alter the synaptic function in female mouse brain. Figure S3. CSF1R haploinsufficiency results in noteworthy changes in the enrichment gene sets of phagosome or toll-like receptor pathway. Figure S4. CSF1R haploinsufficiency results in enhancement in the mRNA levels of Tnf-α and Il-1β both in vivo and in vitro. Figure S5. CSF1R haploinsufficiency does not affect the phagocytosis of microsphere beads by microglia. Figure S6. Minocycline exposure partially inhibits the mRNA levels of Tnf-α and Il-1β in Csf1r+/— microglia or Csf1r+/— mouse brain. Figure S7. The density of microglia is reduced in Csf1r+/— mouse brain. Figure S8. Astrocyte is activated in Csf1r+/— mouse brain. [file 12974_2023_2774_MOESM7_ESM.zip › New folder/Supplementary Figure 2.tif]

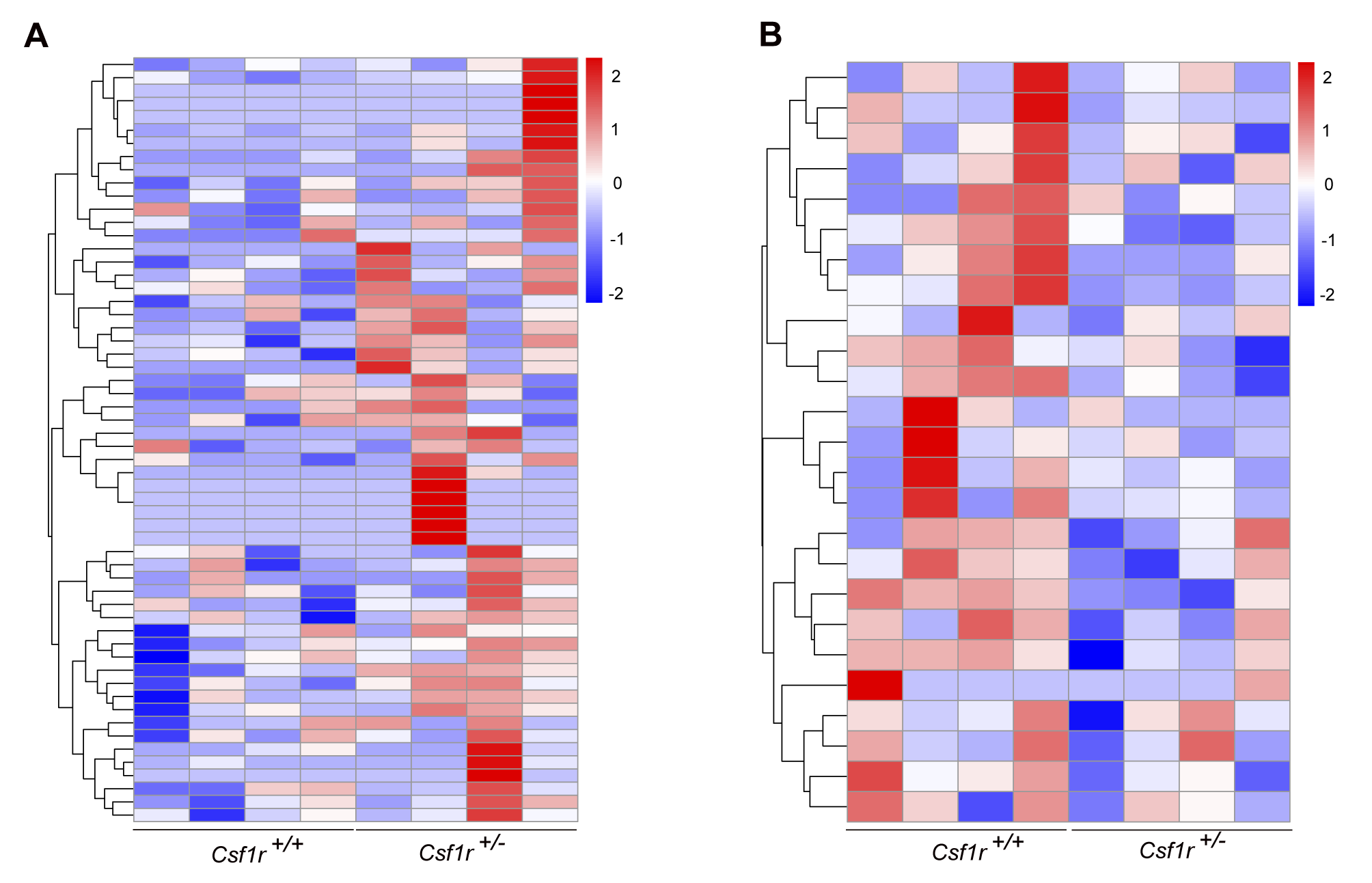

Supplement: Supplementary file 7 — Additional file 7: Figure S1. Identification of CSF1R expression in Csf1r+/— microglia and Csf1r+/− mouse brain. Figure S2. CSF1R haploinsufficiency does not alter the synaptic function in female mouse brain. Figure S3. CSF1R haploinsufficiency results in noteworthy changes in the enrichment gene sets of phagosome or toll-like receptor pathway. Figure S4. CSF1R haploinsufficiency results in enhancement in the mRNA levels of Tnf-α and Il-1β both in vivo and in vitro. Figure S5. CSF1R haploinsufficiency does not affect the phagocytosis of microsphere beads by microglia. Figure S6. Minocycline exposure partially inhibits the mRNA levels of Tnf-α and Il-1β in Csf1r+/— microglia or Csf1r+/— mouse brain. Figure S7. The density of microglia is reduced in Csf1r+/— mouse brain. Figure S8. Astrocyte is activated in Csf1r+/— mouse brain. [file 12974_2023_2774_MOESM7_ESM.zip › New folder/Supplementary Figure 3.tif]

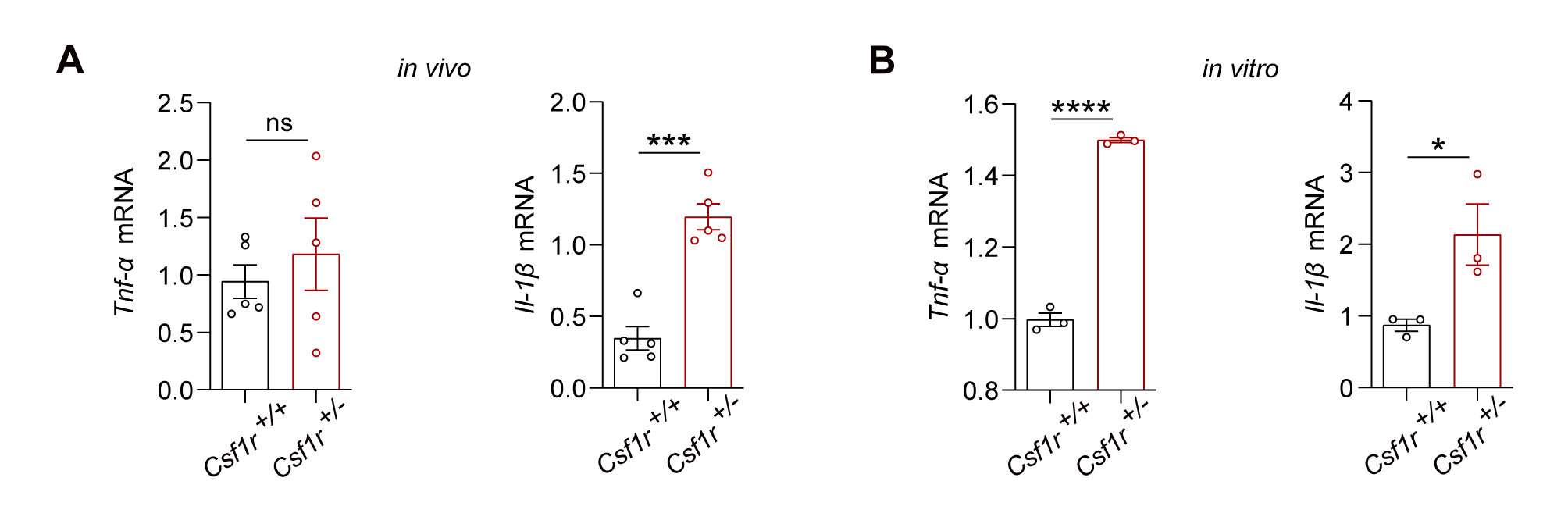

Supplement: Supplementary file 7 — Additional file 7: Figure S1. Identification of CSF1R expression in Csf1r+/— microglia and Csf1r+/− mouse brain. Figure S2. CSF1R haploinsufficiency does not alter the synaptic function in female mouse brain. Figure S3. CSF1R haploinsufficiency results in noteworthy changes in the enrichment gene sets of phagosome or toll-like receptor pathway. Figure S4. CSF1R haploinsufficiency results in enhancement in the mRNA levels of Tnf-α and Il-1β both in vivo and in vitro. Figure S5. CSF1R haploinsufficiency does not affect the phagocytosis of microsphere beads by microglia. Figure S6. Minocycline exposure partially inhibits the mRNA levels of Tnf-α and Il-1β in Csf1r+/— microglia or Csf1r+/— mouse brain. Figure S7. The density of microglia is reduced in Csf1r+/— mouse brain. Figure S8. Astrocyte is activated in Csf1r+/— mouse brain. [file 12974_2023_2774_MOESM7_ESM.zip › New folder/Supplementary Figure 4.tif]

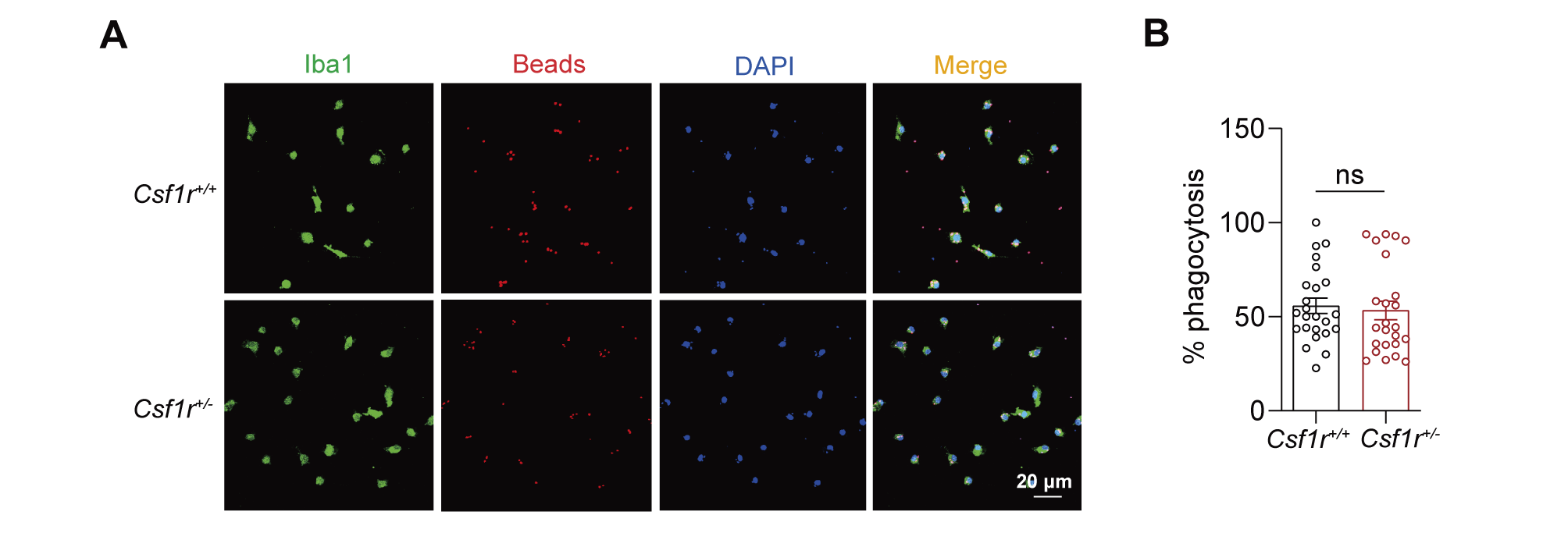

Supplement: Supplementary file 7 — Additional file 7: Figure S1. Identification of CSF1R expression in Csf1r+/— microglia and Csf1r+/− mouse brain. Figure S2. CSF1R haploinsufficiency does not alter the synaptic function in female mouse brain. Figure S3. CSF1R haploinsufficiency results in noteworthy changes in the enrichment gene sets of phagosome or toll-like receptor pathway. Figure S4. CSF1R haploinsufficiency results in enhancement in the mRNA levels of Tnf-α and Il-1β both in vivo and in vitro. Figure S5. CSF1R haploinsufficiency does not affect the phagocytosis of microsphere beads by microglia. Figure S6. Minocycline exposure partially inhibits the mRNA levels of Tnf-α and Il-1β in Csf1r+/— microglia or Csf1r+/— mouse brain. Figure S7. The density of microglia is reduced in Csf1r+/— mouse brain. Figure S8. Astrocyte is activated in Csf1r+/— mouse brain. [file 12974_2023_2774_MOESM7_ESM.zip › New folder/Supplementary Figure 5.tif]

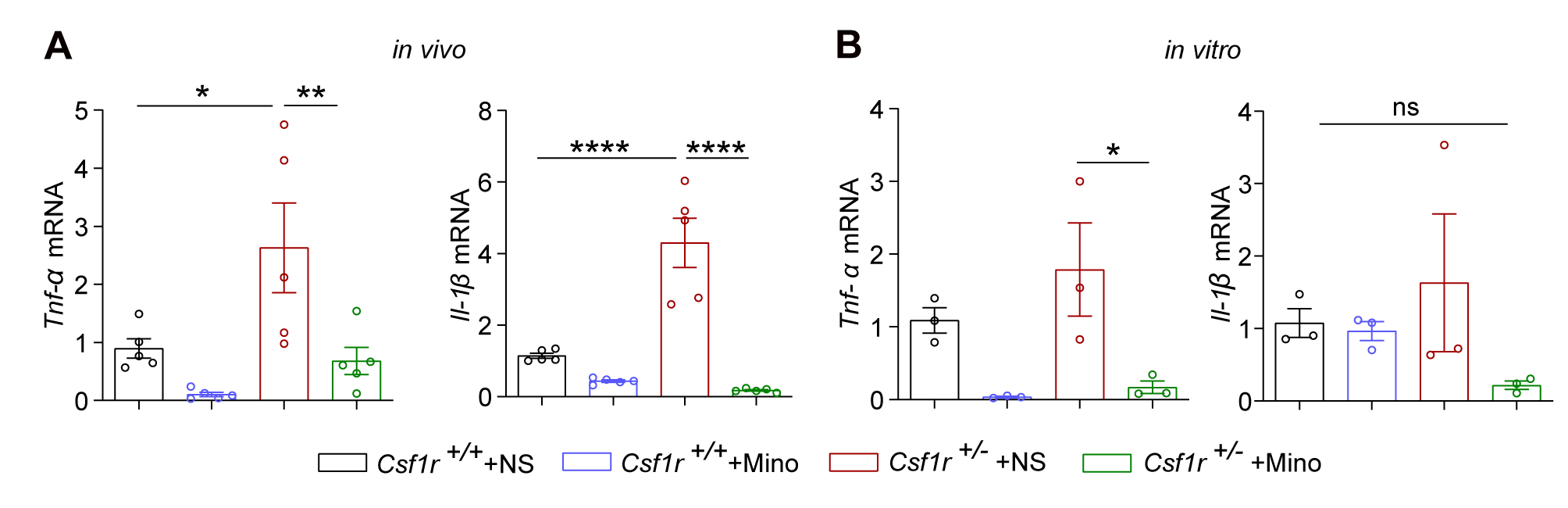

Supplement: Supplementary file 7 — Additional file 7: Figure S1. Identification of CSF1R expression in Csf1r+/— microglia and Csf1r+/− mouse brain. Figure S2. CSF1R haploinsufficiency does not alter the synaptic function in female mouse brain. Figure S3. CSF1R haploinsufficiency results in noteworthy changes in the enrichment gene sets of phagosome or toll-like receptor pathway. Figure S4. CSF1R haploinsufficiency results in enhancement in the mRNA levels of Tnf-α and Il-1β both in vivo and in vitro. Figure S5. CSF1R haploinsufficiency does not affect the phagocytosis of microsphere beads by microglia. Figure S6. Minocycline exposure partially inhibits the mRNA levels of Tnf-α and Il-1β in Csf1r+/— microglia or Csf1r+/— mouse brain. Figure S7. The density of microglia is reduced in Csf1r+/— mouse brain. Figure S8. Astrocyte is activated in Csf1r+/— mouse brain. [file 12974_2023_2774_MOESM7_ESM.zip › New folder/Supplementary Figure 6.tif]

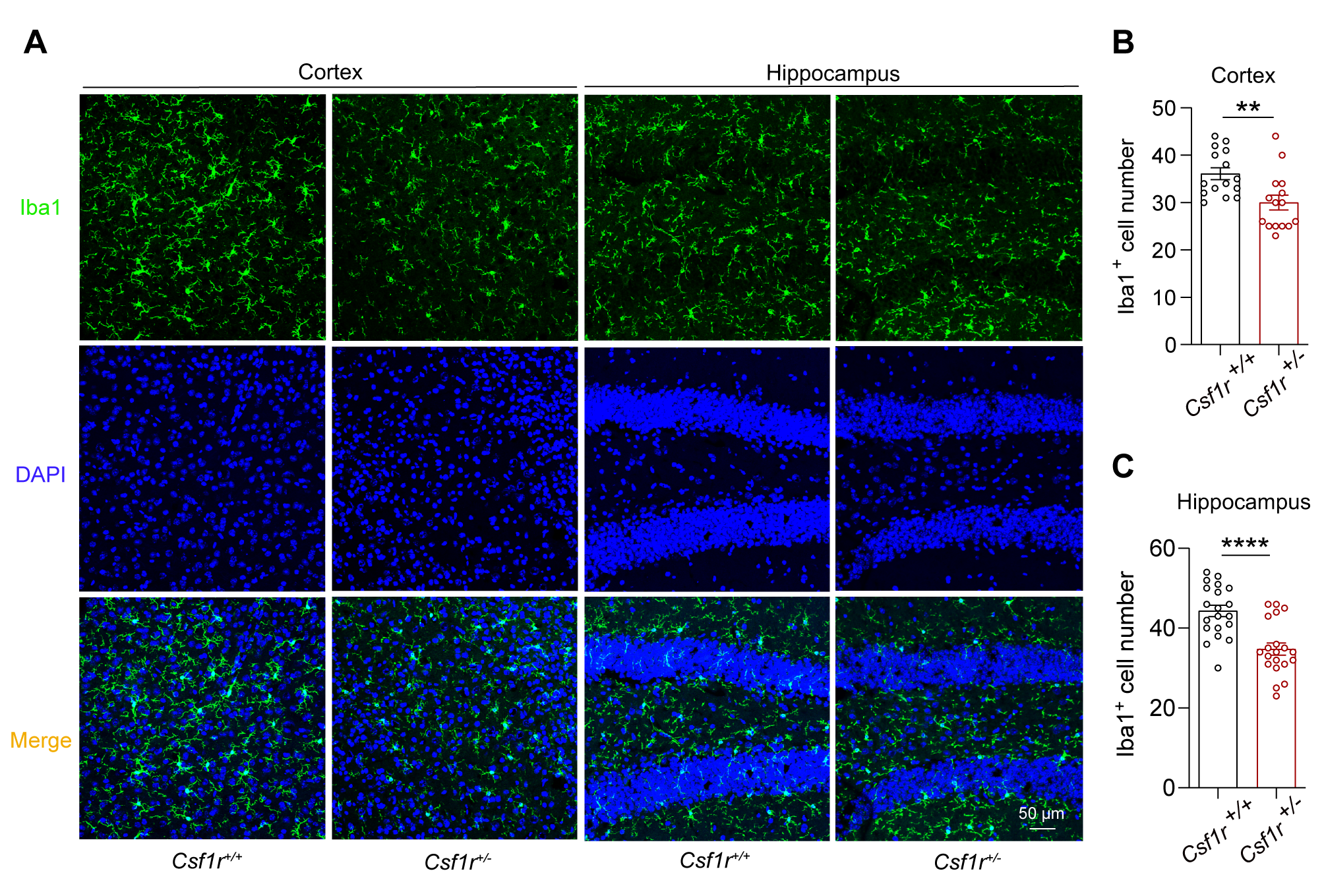

Supplement: Supplementary file 7 — Additional file 7: Figure S1. Identification of CSF1R expression in Csf1r+/— microglia and Csf1r+/− mouse brain. Figure S2. CSF1R haploinsufficiency does not alter the synaptic function in female mouse brain. Figure S3. CSF1R haploinsufficiency results in noteworthy changes in the enrichment gene sets of phagosome or toll-like receptor pathway. Figure S4. CSF1R haploinsufficiency results in enhancement in the mRNA levels of Tnf-α and Il-1β both in vivo and in vitro. Figure S5. CSF1R haploinsufficiency does not affect the phagocytosis of microsphere beads by microglia. Figure S6. Minocycline exposure partially inhibits the mRNA levels of Tnf-α and Il-1β in Csf1r+/— microglia or Csf1r+/— mouse brain. Figure S7. The density of microglia is reduced in Csf1r+/— mouse brain. Figure S8. Astrocyte is activated in Csf1r+/— mouse brain. [file 12974_2023_2774_MOESM7_ESM.zip › New folder/Supplementary Figure 7.tif]

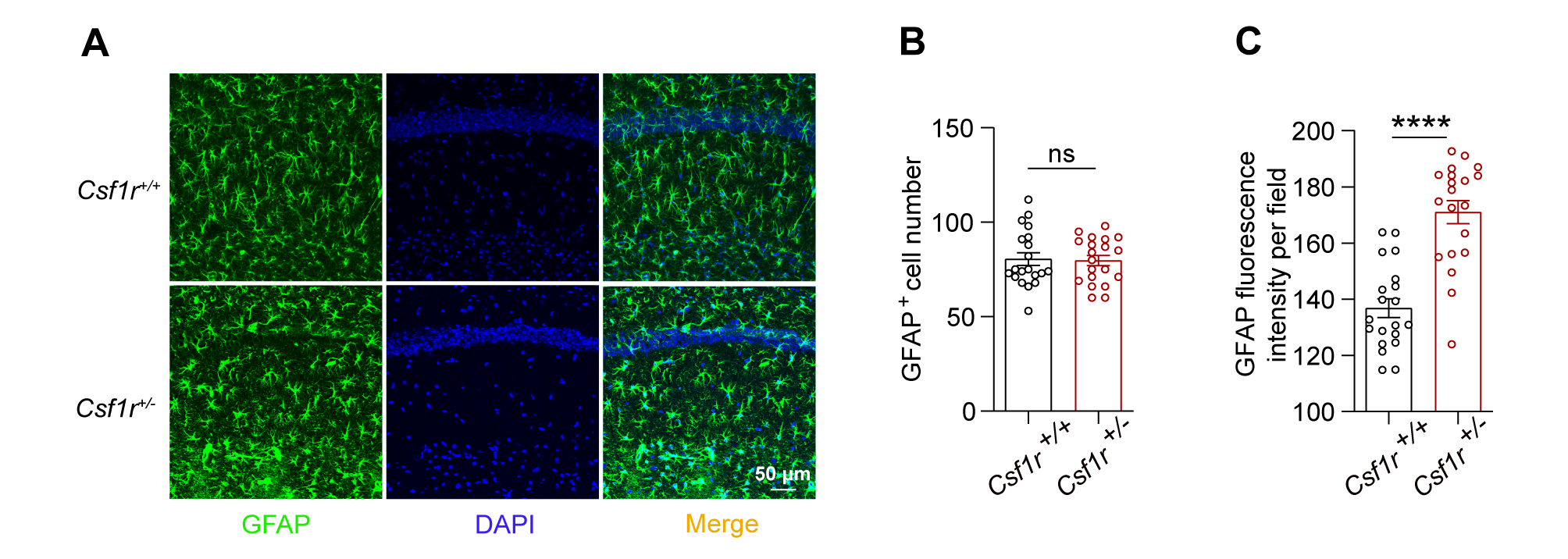

Supplement: Supplementary file 7 — Additional file 7: Figure S1. Identification of CSF1R expression in Csf1r+/— microglia and Csf1r+/− mouse brain. Figure S2. CSF1R haploinsufficiency does not alter the synaptic function in female mouse brain. Figure S3. CSF1R haploinsufficiency results in noteworthy changes in the enrichment gene sets of phagosome or toll-like receptor pathway. Figure S4. CSF1R haploinsufficiency results in enhancement in the mRNA levels of Tnf-α and Il-1β both in vivo and in vitro. Figure S5. CSF1R haploinsufficiency does not affect the phagocytosis of microsphere beads by microglia. Figure S6. Minocycline exposure partially inhibits the mRNA levels of Tnf-α and Il-1β in Csf1r+/— microglia or Csf1r+/— mouse brain. Figure S7. The density of microglia is reduced in Csf1r+/— mouse brain. Figure S8. Astrocyte is activated in Csf1r+/— mouse brain. [file 12974_2023_2774_MOESM7_ESM.zip › New folder/Supplementary Figure 8.tif]
